# Efficacy of a Contact Lens Sensor for Monitoring 24-H Intraocular Pressure Related Patterns

**DOI:** 10.1371/journal.pone.0125530

**Published:** 2015-05-05

**Authors:** Kaweh Mansouri, Robert N. Weinreb, John H. K. Liu

**Affiliations:** 1 Hamilton Glaucoma Center and Department of Ophthalmology, University of California San Diego, La Jolla, California, United States of America; 2 Glaucoma Sector, Department of Ophthalmology, Geneva University Hospitals, Geneva, Switzerland; Sun Yat-sen University, CHINA

## Abstract

**Purpose:**

To study performance of a contact lens sensor (CLS) for 24-hour monitoring of IOP-related short-term patterns and compare with IOP obtained by pneumatonometry.

**Methods:**

Prospective clinical trial. Thirty-one healthy volunteers and 2 glaucoma patients were housed for 24 hours in a sleep laboratory. One randomly selected eye was fitted with a CLS (Triggerfish, Sensimed, Switzerland), which measures changes in ocular circumference. In the contralateral eye, IOP measurements were taken using a pneumatonometer every two hours with subjects in the habitual body positions. Heart rate (HR) was measured 3 times during the night for periods of 6 minutes separated by 2 hours. Performance of CLS was defined in two ways: 1) recording the known pattern of IOP increase going from awake (sitting position) to sleep (recumbent), defined as the wake/sleep (W/S) slope and 2) accuracy of the ocular pulse frequency (OPF) concurrent to that of the HR interval. Strength of association between overall CLS and pneumatonometer curves was assessed using coefficients of determination (R^2^).

**Results:**

The W/S slope was statistically significantly positive in both eyes of each subject (CLS, 57.0 ± 40.5 mVeq/h, p<0.001 and 1.6 ± 0.9 mmHg/h, p<0.05 in the contralateral eye). In all, 87 CLS plots concurrent to the HR interval were evaluated. Graders agreed on evaluability for OPF in 83.9% of CLS plots. Accuracy of the CLS to detect the OPF was 86.5%. Coefficient of correlation between CLS and pneumatonometer for the mean 24-h curve was R^2^ = 0.914.

**Conclusions:**

CLS measurements compare well to the pneumatonometer and may be of practical use for detection of sleep-induced IOP changes. The CLS also is able to detect ocular pulsations with good accuracy in a majority of eyes.

**Trial Registration:**

ClinicalTrials.gov NCT01390779

## Introduction

Lowering intraocular pressure (IOP) is currently the only proven method for preventing or reducing the development and progression of glaucoma. [1] Therefore, accurate assessment of IOP represents the cornerstone of glaucoma management. Despite the understanding that IOP is a highly dynamic parameter, [2] it is commonly assessed only during office hours by static tonometry techniques, with point-in-time measurements. Consequently, the real diurnal IOP and, potentially as important, the nocturnal IOP remain unknown. [3] This shortcoming in the assessment of the main risk factor for glaucoma may underlie the progressive glaucomatous optic neuropathy observed in some patients who have office-hour IOP within the designated target range. [4] In the absence of a practical tool for continuous 24-h IOP monitoring, alternative strategies, such as provocative testing [5] and diurnal tension curves [6] have been proposed. However, none of these provides an accurate estimation of the 24-hour IOP behavior. [3]

To address this unmet need, a contact lens sensor (CLS) has been developed with the intention of monitoring 24-hour IOP in ambulatory conditions. [7,8] Several reports have demonstrated good safety and tolerability of the CLS for 24-hour use [9, 10] as well as good reproducibility of measurements. [11–13] The assumption behind this technology is that variations in IOP lead to changes in ocular volume and dimensions, which the CLS captures through embedded strain gauges. Although this assumption has been validated in vitro, [7] the practical use of CLS data needs to be validated. Since the CLS output signal is provided in relative units (that correspond to electrical units of voltage) and tonometry is provided in absolute mmHg units, a direct comparison between the two methods cannot be performed. This difficulty is further compounded by the inability to use tonometry simultaneously on the CLS-wearing eye.

One way to circumvent this problem is to use the fellow eye as a comparator. For this purpose, a strong correlation between the two eyes would be required. [14, 15] Studies show that IOP may fluctuate moderately in parallel in healthy fellow eyes whereas IOP peaks may not appear at the same time for an individual subject. [15] The concordance of diurnal IOP between fellow eyes with glaucoma was evaluated in one study which found a 68% to 90% probability for the absolute change in IOP between fellow eyes to be within 2 mmHg and 78% to 95% within 3 mmHg. [16] Others, however, previously reported a weaker relationship, with correlations between fellow eyes ranging between 0.65 and 0.73 (mean r = 0.70) under various conditions. [17] Additionally, about 85% of fellow eye IOPs were within 3 mmHg at any given measurement time point. Of course, when IOP-related data are collected in paired eyes by different methods, technical factors may have a pronounced effect on the estimation of 24-hour IOP patterns compared to the use of a single method.

The aim of this trial was to assess performance of the CLS by correlating its output with a systemic parameter (heart rate) and IOP obtained with the pneumatonometer. For this purpose, two parameters were identified which represent both short duration (seconds) and longer duration (hours) IOP-related patterns. The first, ocular pulse frequency (OPF) corresponds to IOP variations due to systole and diastole during the cardiac cycle. [18] The second, the wake/sleep slope (W/S) is a CLS-derived parameter which quantifies the characteristic IOP rise that occurs when subjects go from the wake/sitting to the sleep/supine state. [11] Both parameters can be derived independently of mmHg and thus overcome the main shortcoming of the CLS.

## Methods

The protocol for this trial and supporting CONSORT checklist are available as supporting information; see S1 CONSORT Checklist and S1 Protocol.

This was a single-center prospective clinical trial (clinicaltrials.gov; NCT01390779) with subjects serving as their own control. It was compliant with the Health Insurance Portability and Accountability Act. All research involving human participants were approved by the University of California, San Diego Institutional Review Board (IRB), and all clinical investigation were conducted according to the principles expressed in the Declaration of Helsinki. Written informed consent was obtained from the participants.

All subjects underwent the same study procedures which consisted of a complete ophthalmic examination consisting of a medical history, best-corrected visual acuity, refraction, ocular biometry, central corneal thickness using an ultrasonic pachymeter (Pachette 2; DGH Technology, Exton, PA), slit-lamp biomicroscopy, dilated ophthalmoscopy, and Goldmann applanation tonometry (Haag-Streit, Mason, OH) during office hours. Healthy subjects, aged between 18 and 80 years of either sex, including ocular hypertension or patients with primary open angle glaucoma (POAG) were eligible for participation in the study. The use of IOP-lowering medications was not permitted; if used, they were washed out for at least 4 weeks prior to participation in the study. A difference in IOP of less than 3 mmHg between the two eyes was required in an attempt to minimize inter-eye differences. In addition, the following inclusion criteria were applicable: best corrected visual acuity of ≥ 20/80 in both eyes and open angles. Exclusion criteria included the presence of ocular disease other than primary open-angle glaucoma, spherical equivalent more than 4 diopters, a cylinder equivalent more than 2 diopters, and corneal or conjunctival abnormalities hindering adaptation of silicon contact lens.

Laboratory conditions were controlled as described previously. [19] Enrolled subjects were required to have a regular daily sleep cycle of approximately 8 hours. Prior to study start, they were instructed to maintain an accustomed 8-hour sleep for 7 days. Subjects were given a wrist-mounted device (Actiwatch; Mini Mitter, Sunriver, OR) and a wake/sleep log to keep track of physical activity and light exposure. Subjects were further provided with a diary for recording of physical activities, intake of meals and medications, emotional status, and other events every half hour in the sleep laboratory. The 8-hour period of darkness in each sleep room was adjusted close to the individual’s sleep cycle, and times for the IOP measurements in the contralateral eye were individualized correspondingly. For data presentation, clock times for lights off (between 10 PM and 11:30 PM) were normalized as if each subject slept from 11 PM to 7 AM.

Subjects presented at the study site for placement of the CLS in one randomly selected eye. After successful CLS fitting and data recording initiation at 2 PM ± 2 hours, the study was carried out in the adjacent sleep laboratory for the subsequent 24-hour period. Measurements of IOP in the contralateral eye were taken every 2 hours by experienced technicians using a calibrated pneumatonometer (Model 30 Classic; Reichert, Depew, NY). Measurements were taken in the habitual body positions (sitting during the diurnal/wake period and supine during the nocturnal/sleep period). Before the nocturnal/sleep period, IOP was measured at 3:30 PM, 5:30 PM, 7:30 PM, and 9:30 PM. Subjects were instructed to sit for 5 minutes. Blood pressure and heart rate were measured immediately before the IOP measurements. Lights were turned off at 11:00 PM and nocturnal measurements were taken at 11:30 PM, 1:30 AM, 3:30 AM, and 5:30 AM. Subjects were awakened, if necessary, and the measurements of blood pressure, heart rate, and IOP were taken supine under dim light (<10 lux). Light exposure was kept to a minimum during the nocturnal period. Three times during the sleep period, heart rate (HR) was measured directly over periods of six minutes each. This duration was chosen since CLS activates every five minutes for 30 seconds, and time of activation cannot be known until device recordings are examined. Thus, the interval specified ensured at least one continuous thirty-second CLS recording in parallel to each HR measurement. When the sleep period ended at 7 AM, room lights were turned on and subjects were awakened, if necessary. Diurnal measurements were taken again at 7:30 AM, 9:30 AM, 11:30 AM, and 1:30 PM as described previously. After completion of the 24-h recording, the CLS was removed from the subject, the activity diary was collected, and an ophthalmologic examination including the measurement of central corneal thickness was performed. Any change from baseline in ocular parameters, as assessed by the investigator or the patient, was defined as an adverse event.

### Instruments

The CLS (SENSIMED Triggerfish, Sensimed, Switzerland) measures spontaneous dimensional changes of the eye at the corneoscleral junction to record the IOP-related profile for 24 hours in the habitual position. Over 24 hours, the CLS activates during 288 data recording periods of 30 seconds each, which are provided in mV equivalents (mVeq). The data are continuously transmitted wirelessly via a patched antenna around the orbit and connected to a data recorder. Upon completion of the recording session, data are downloaded to a computer for visualization of the 24-hour IOP-related profile, including long-term (e.g. W/S slope) and short-term (e.g. ocular pulse) patterns. The device is not approved for the market in the USA, but is considered non-significant risk for clinical study conduct by FDA. The device has been described in more detail elsewhere. [20]

### Primary Outcome Measures

In order to demonstrate the CLS’ ability to record IOP-related patterns, two instances of such patterns have been selected a priori as co-primary efficacy endpoints, the OPF and the W/S slope. The OPF, a short duration pattern, was defined as the difference between frequency of ocular pulsations as recorded by the CLS and the heart rate as determined by pulse frequency assessment, its agreement being scored dichotomously at each pair of parallel measurements. Manual evaluation of 30 sec. CLS plots to assess the OPF was done by two independent and masked graders. The data points closest to the three 6 min. intervals at which HR was assessed during the night were selected for the comparison. The mean of the graders’ results for each plot served as the 30-second OPF. Data were included in the analysis when either one or both graders judged the signal to be evaluable for assessment of OPF and provided an estimate of OPF. The W/S slope, a longer duration IOP-related pattern was defined as the CLS output from 1 hour preceding the time of dark period initiation (going to bed) to 1 hour after. [11]

### Statistical Analysis

The hypothesis for the study was that the lower margin of the confidence interval of the accuracy of the CLS to detect OPF would be greater than or equal to 70% (H_0_: π_accuracy_ < 0.70 and H_1_: π_accuracy_ ≥ 0.70). The π_accuracy_ is defined as the proportion of ocular pulse values for one minute intervals as recorded by the CLS that are within ±15% of HR measured directly. As planned, this co-primary outcome measure was tested by constructing a 95% two-sided confidence interval using SAS PROC GENMOD (logistic link function) to account for possible dependency between measurements taken from the same individual. The second hypothesis for the study was that a positive W/S slope would be detected. For this reason, only subjects showing an IOP increase of at least 3 mmHg on pneumatonometry from wake to sleep were included in the analysis. The model used was IOP (measured by CLS) as a function of time (independent variable) within each subject; i.e. each subject’s slope was estimated in a separate analysis. Repeated measures could not be accounted for since analysis was done within subject (so that there can be no distinction between within and between variance). In any event, it is important to note that our interest is in each subject’s slope and not in the variation about the slope. The mean of the independent subject slopes is then tested for its difference from 0. Thus, the variation of that test is based on a single (slope) measurement for each subject and no repeated measurement is involved. The W/S slope was analyzed (H_0_: W/S slope = 0, H_1_: W/S slope > 0), using SAS PROC GLM within the specified time window. We used a general linear model allowing the mean to depend on a linear predictor through a logistic (nonlinear, binary) function, where the dependent binary outcome is repeated. Specifically, the dependent variable was agreement/disagreement between CLS measured OPF and manually measured OPF, measured repeatedly (within subject), with the independent variable being subject (categorical). Correlation was assumed to be unstructured. The confidence interval was tested using the T-test. In addition, the strength of association between overall CLS and pneumatonometer curves was calculated using coefficients of determination (R^2^). For this analysis, only the average CLS values corresponding to the two-hourly pneumatonometry readings were retained.

Power analyses were based on assumptions derived from earlier studies with CLS and was done for the two endpoints separately. For OPF, given 33 subjects, we expected at least N = 30 valid and independent OPF measurement pairs (by CLS and HR measurement). Assuming OPF agreement of 90%, a sample of 30 independent measurements had slightly over 80% power to demonstrate an agreement of at least 70%. Power was likely higher given multiple measurements per subject, a simulation that was not conducted because the assumption of independence coupled with 30 observations provided sufficient power for testing this hypothesis. Coupled with the power of the second hypothesis described below, the power for the trial as a whole was about 80%, assuming that OPF and W/S are independent. For W/S, assuming an average slope of 60 mVeq/h ± 50 mVeq/h (for subjects with positive W/S measured by tonometry), as sample of 30 subjects has virtually 100% power to detect a slope that is greater than 0. It should be noted that in this case observations are independent since each subject provides a single slope only.

## Results

Thirty-three individuals (31 healthy subjects and 2 glaucoma patients) were enrolled in this clinical trial. Seventeen individuals (51.5%) had the left eye selected for CLS wear. **Table 1** summarizes baseline demographic and ocular characteristics during office hours of the entire study population. The intraclass correlation coefficient for IOP in both eyes was 0.875. Comparing the two sets of eyes with CLS or pneumatonometry, there was no difference in ocular parameters. The average length of use of the CLS was 24.1 ± 0.05 hours. The study population for the safety analysis included all 33 participants. The investigators attributed 42% (24 events in 13 participants) of adverse events to the device. The most frequently reported events were hyperemia of the bulbar and palpebral conjunctiva (8 cases) and superficial punctuate keratitis (7 cases). Adverse events were transient and resolved within 48 hours after CLS removal. There were no serious adverse events and no safety issues in the fellow eye.

**Table 1:** Baseline Demographic and Ocular Characteristics of Study Participants (n = 33).

Age, yrs (mean ± SD)	35.0 ± 14.4
Gender	
Male	17 (51.5%)
Female	16 (48.5%)
Ancestry	
Caucasian	26 (78.8%)
Asian	6 (18.2%)
African-American	1 (3.0%)
SER, D (mean ± SD)	
CLS eye	-0.7 ± 1.3
Pneumatonometer eye	-0.8 ± 1.2
CCT, μm (mean ± SD)	
CLS eye	560.3 ± 42.4
Pneumatonometer eye	560.6 ± 41.1
IOP, mmHg (mean ± SD)	
CLS eye	17.8 ± 2.8
Pneumatonometer eye	17.8 ± 2.9
Body Mass Index (kg/m^2^), (mean ± SD)	25.0 ± 4.1

Abbreviations: CCT = Central corneal thickness; CLS = Contact lens sensor; SER = Spherical equivalent refraction

**Fig 1:**
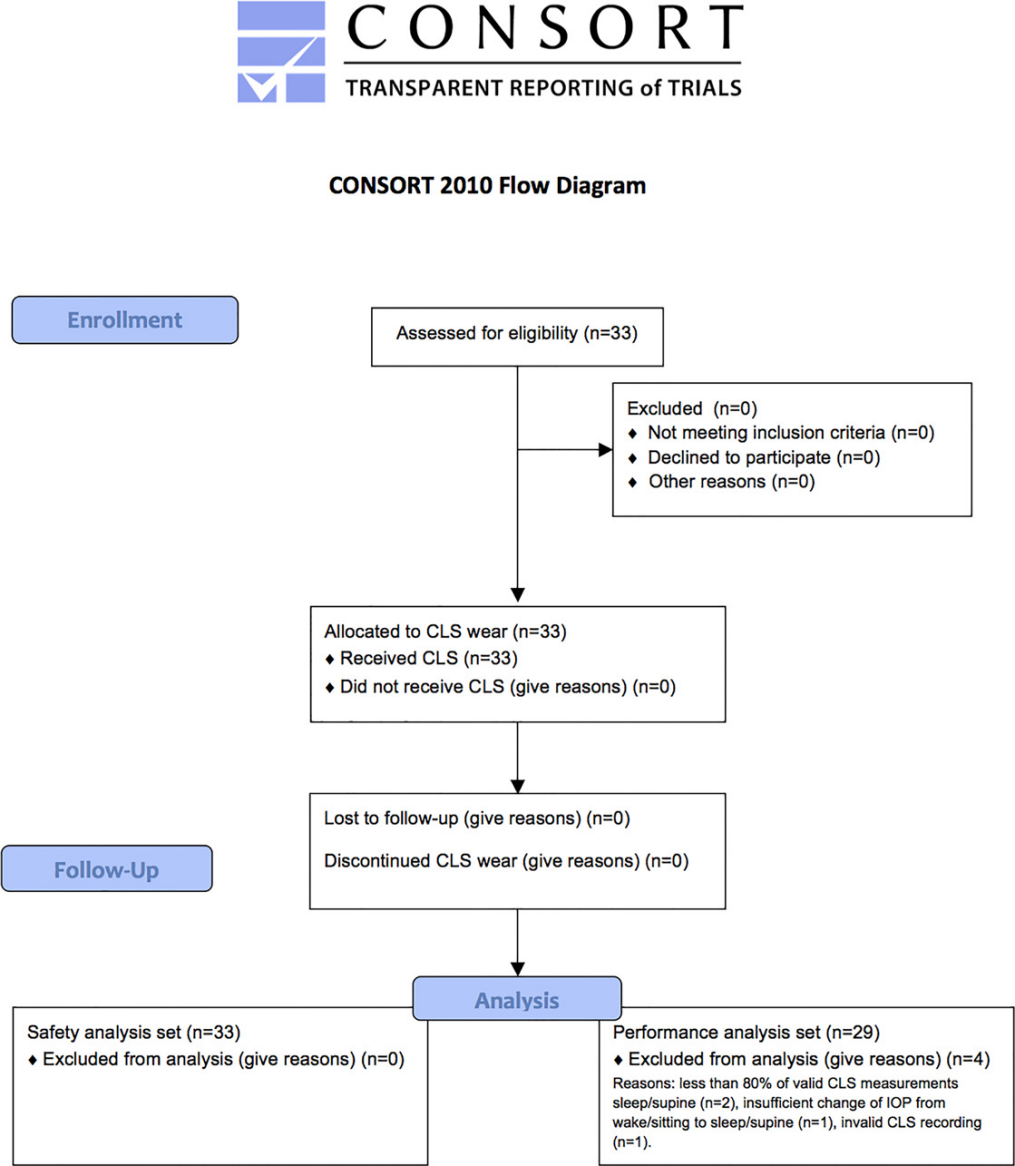
CONSORT flow chart.

In accordance with study protocol, 4 individuals were excluded from the CLS performance analysis. The exclusions were made for the following reasons: 1) less than 80% of valid CLS measurements were obtained within one hour before/after dark period (n = 2), 2) change of IOP from wake/sitting to sleep/supine as measured by pneumatonometry was less than 3 mmHg (n = 1), and 3) invalid CLS recording (n = 1). Patient flow is shown in Fig 1.

**Fig 2:**
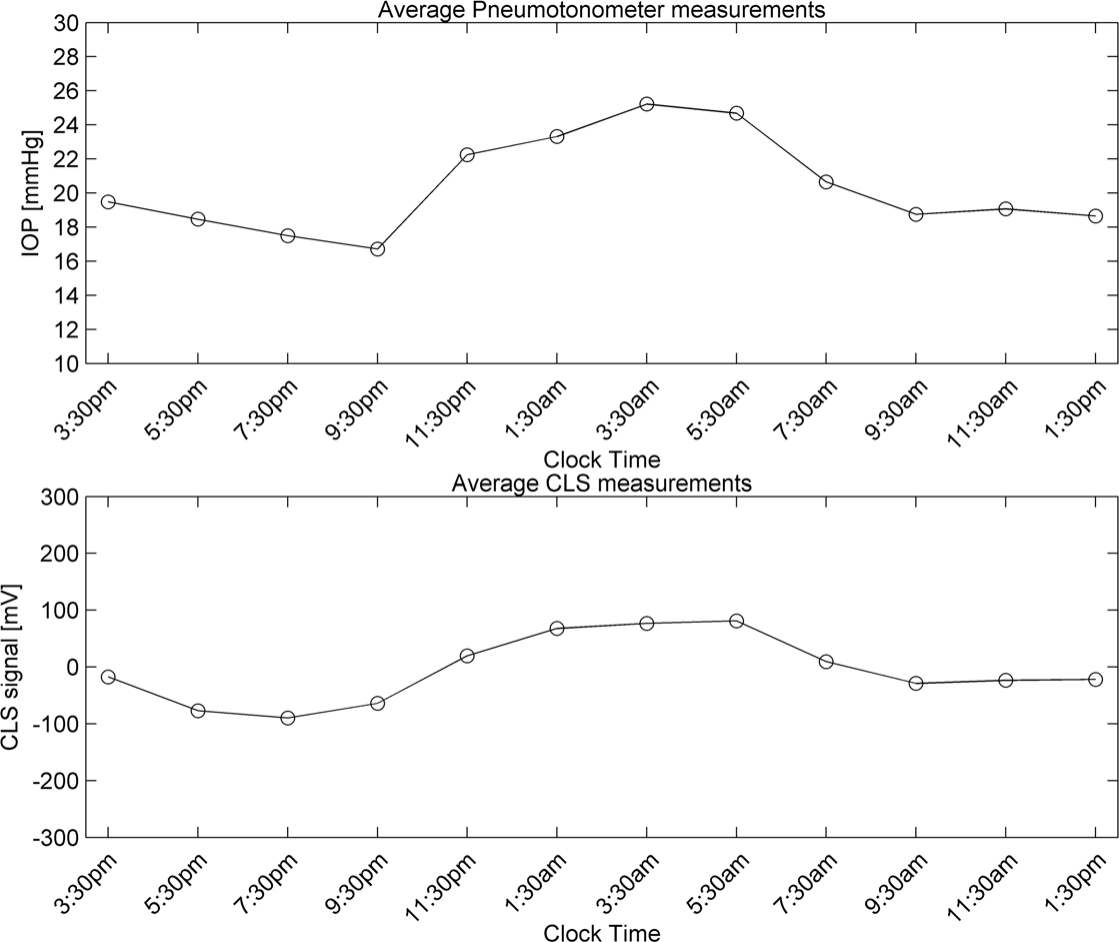
Twenty-four-hour IOP pattern measured in CLS (1B) and pneumatonometer eye (1A) (N = 30). Coefficient of determination between the two eyes was R^2^ = 0.956. Pneumatonometer data were collected sitting during the diurnal/wake period and supine during the nocturnal/sleep period.

Central corneal thickness (CCT) decreased from 560.3 ± 42.4 μm before CLS placement to 548.0 ± 46.5 μm after CLS removal at 24 hours (p < 0.001). In the fellow eye, CCT was 560.6 ± 41.1 μm and 559.9 ± 44.0 μm, respectively (p = 0.182). Overall, there was high correlation between the mean CLS curve and the pneumatonometer curve at two-hourly time-points (R^2^ = 0.914, p < 0.001; Pearson correlation coefficient). (Fig 2)

**Fig 3:**
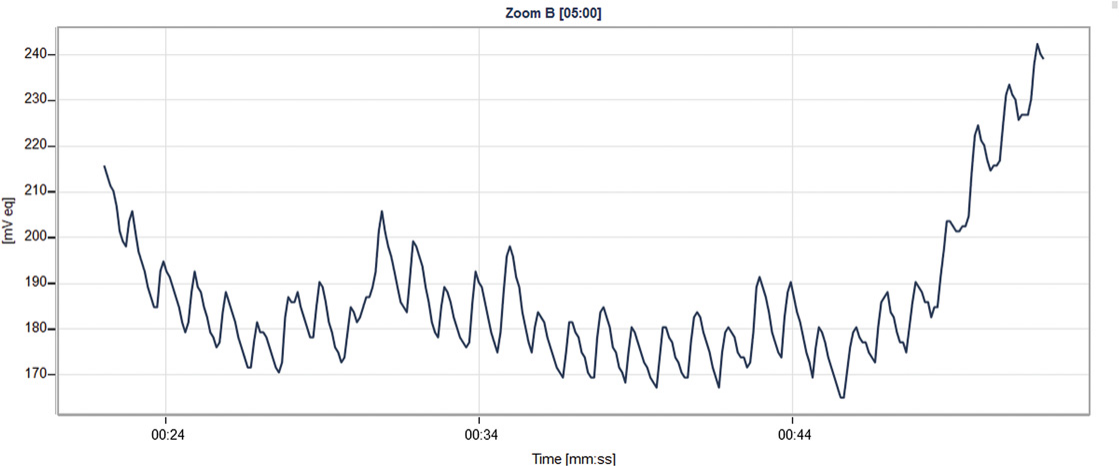
Example of 30-second plot with the CLS (contact lens sensor) with the patient asleep. The oscillating signal represents diastolic to systolic variations in IOP due to heart activity, referred to as OPF.

Two independent graders evaluated a total of 87 CLS plots that corresponded to the HR interval of 29 subjects for determination of OPF. **Fig 3** provides an example of a 30-sec CLS plot. The graders agreed on whether or not the CLS output was evaluable for OPF in 83.9% of cases (43.7% and 40.2% agreeing on evaluable and non-evaluable, respectively), whereas there was no agreement for 16.1% of plots. The accuracy of the CLS to detect the OPF was 86.5%, with the lower margin of the estimated 95% CI being 75.0%, thus rejecting the null hypothesis. For an α of 0.0475 (type I error), the difference between the HR and the estimated OPF is smaller than 15% in at least 70% of evaluable cases. To assess the potential effect of awakening on the CLS signal, a post-hoc analysis was performed in which the first and second CLS data points (T1 and T2) preceding per protocol CLS data point (T0), were used. As shown in Table 2, the CLS plots preceding the awakening of patients for HR measurement, did not differ in accuracy. Therefore, the waking up of subjects had no statistically significant effect on this outcome measure.

**Table 2:** Accuracy of ocular pulse frequency by contact lens sensor in relation to heart rate interval.

OPF interval	Proportion of accurate cases	Lower 95.25% CI	Upper 95.25% CI
T0(per protocol)	86.5%(45 out of 52 plots)	75.0%	93.2%
T1	85.5%(49 out of 56 plots)	64.1%	95.1%
T2	86.0%(58 out of 68 plots)	74.5%	92.9%

Abbreviations: CI = confidence interval; OPF = ocular pulse frequency

The analysis includes OPF intervals T1 and T2, which refer to the two 30-second CLS recording intervals immediately preceding the time of the heart rate assessment (T0). The column “proportion of accurate cases” was determined using the average of the two graders’ evaluations.

**Fig 4:**
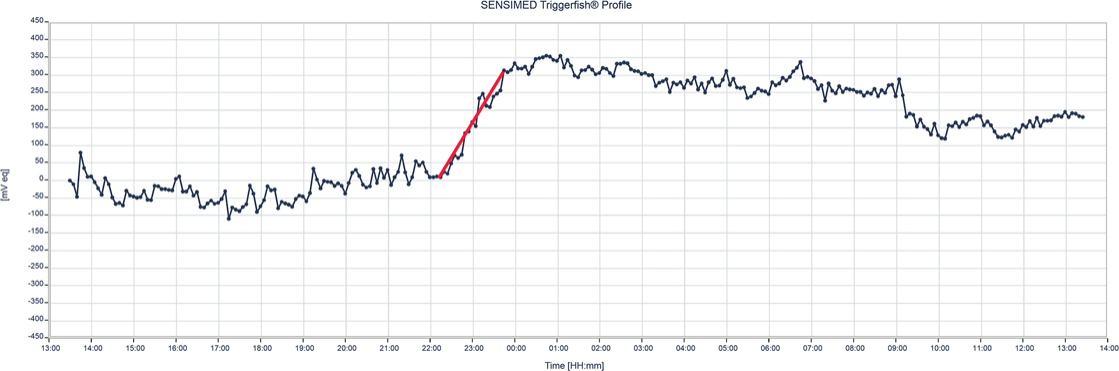
Example of 24-h CLS pattern recorded in a study subject. The red line represents the wake/sleep (W/S) slope as determined from 1 hour pre- to 1 hour post-sleep.

**Fig 5:**
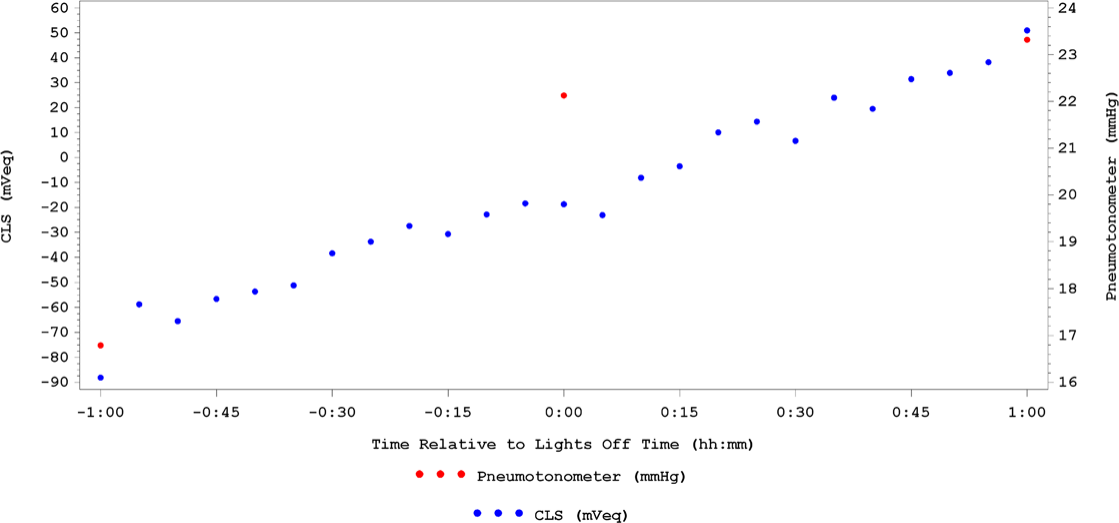
Calculation of wake/sleep (W/S) slope as determined from 1 hour pre- to 1 hour post-sleep with CLS (blue). For the pneumatonometer means (red), actual relative time is not used, but rather one point before, one point closely following and one point after “lights off time”, with a two hour difference between each.

In the CLS eye, the W/S slope was statistically significantly positive (57.0 ± 40.5 mVeq/h; 95% CI, 41.6–72.4 mVeq/h, p<0.001) and the null hypothesis was rejected using the final α of 0.0475. Fig 4 shows an example of the W/S slope in one patient. Calculation of the W/S slope is depicted in Fig 5.

All but one subject (3.4%) had a positive W/S slope, which did not have an impact on the significance of the outcome. In the pneumatonometer eye, a positive W/S slope (1.6 ± 0.9 mmHg/h, p<0.05) was found based on 3 IOP readings for each subject.

## Discussion

In the present study, we show that the CLS can accurately detect ocular pulsations that occur simultaneous to the heart rate. The current CLS software, however, is not well suited for detection of ocular pulsations, which are graphically identifiable as regular low-amplitude oscillations (Fig 2). These are largely overshadowed by more prominent signal spikes that are due to eye blinks and saccades. [21, 22] Therefore, ocular pulsations are mostly visible in the sleep period when eyes are mostly closed and eye movements are reduced. In this study, of all CLS outputs, 60% were evaluable for OPF by one or two independent graders. The remainder could not be interpreted by the graders as the output showed erratic peaks and troughs due to eye blinks and movements, thus masking the OPF. This was partially a consequence of awakening subjects for study assessments including heart rate measurement over 6 minutes. Inter-observer agreement on the presence of OPF was 84%. It should be noted that this endpoint was not designed to demonstrate that the CLS could detect the OPF, but to ascertain whether the OPF, when observable, would be comparable to the HR, thus validating the CLS output.

Characteristics of ocular pulsations, such as ocular pulse frequency (OPF) and the ocular pulse amplitude (OPA) have been suggested to be potentially useful clinical parameters. Alterations in ocular blood flow (OBF) have been hypothesized to be related to the pathogenesis of glaucoma. [23, 24] At present, however, no method exists to measure OBF directly and non-invasively. Therefore, attempts have been made to estimate the pulsatile OBF using measurements of the OPA with the dynamic contour tonometer (DCT). [25] This approach has several limitations, including the assumption of an equal pressure-volume relationship among individuals. This assumption may not be valid due to individual differences in ocular biomechanical properties. [26, 27] In addition, DCT does not provide data beyond its 100 second continuous measurement period, data that would be necessary for an adequate 24-h characterization of this parameter, given its dynamic nature and the presence of a circadian rhythm. [28] While some investigators have postulated that the OPA may be an independent risk factor for glaucoma, [29, 30] little is known about the importance of OBF. Use of the CLS to provide 24-h data on the timing, frequency, and amplitude of ocular pulsations may provide information about the complex relationships among ocular blood flow, IOP and glaucoma damage.

We have previously shown that IOP increases when subjects go from sitting/wake to supine/sleep when measured every 2 hours. [31] This study was conducted under the same strict sleep laboratory conditions as described for previous studies. [19] Except for one subject, all other subjects consistently demonstrated a positive W/S slope, despite the IOP-related pattern being unique for each subject. In line with previous investigations, [11] the results in the current study showed the mean W/S slope of the nocturnal CLS pattern was statistically significantly positive in 97% of participants. The pneumatonometer-derived W/S slope, although positive, was less pronounced than the CLS derived slope (Fig 4). There are several reasons for this finding. First, only two hourly measurements were available for the pneumatonometer and, therefore, the W/S slope had to be constructed with only three datapoints over 4 hours. Second, the two device units are not directly comparable, perhaps explaining a major part of the discrepancy. Third, CLS measurements were obtained with eyelids open or closed while participants were awoken for tonometry measurements. Although, Mansouri et al. (unpublished data, clinicaltrials.gov number: NCT01938287) have found no significant effect of eyelid closure on CLS measurements, it cannot be excluded that discrete lid effects may have contributed to the observed difference. Aptel et al., [32] directly addressed the issue of awakening and its effect on IOP measurements. They found that hourly awakening during noncontact tonometer IOP measurements did not seem to alter the mean variables of the 24-hour IOP pattern evaluated using CLS. Fourth, Sit et al., [17] have previously demonstrated that correlation in IOP between fellow eyes was only moderate. Fifth, different measurement principles and their dependency on ocular biomechanical properties may also contribute to the observed differences. Further research is needed to elucidate these questions.

The fact that we could not obtain simultaneous OPF and HR measurements for periods of 30 seconds is a limitation of this study. It is possible that a different accuracy of OPF would have been obtained if longer periods of simultaneous measurements were available. There is a possibility that the act of awakening subjects for HR measurements produced changes in OPF. However, when we analyzed the CLS measurements prior to the concurrent OPF-HR measurement (Table 2, T1 and T2), awakening of subjects for pneumatonometry and HR measurements was found not to have a significant effect on the correlation with OPF assessment. Lastly, a significant reduction of CCT (-12.3 μm in CLS vs. -0.8 μm in fellow eye) in the CLS eye, but not in the fellow eye, was observed. Prior reports have conflicting results with regards to the CLS effect on CCT; some studies reported an increase [11, 33] and others a decrease of CCT. [34] To what extent this change may have affected CLS measurements is currently unknown.

Finally, the study protocol required the inclusion of both healthy subjects and glaucoma patients. However, due to the difficulty of recruiting glaucoma patients with less than 3 mmHg asymmetry between fellow eyes and the need for timely enrollment, only 2 glaucoma patients could be included in this study. Excluding the glaucoma patients from this first study report could have been considered a violation of the obligation for full data reporting as registered on clinicaltrials.gov. Therefore, all study patients were included in the analysis. Although in glaucoma patients with different stages of the disease on right and left eyes, asymmetry of IOP between fellow eyes could potentially affect the comparison of CLS and pneumatonometer measurements of IOP. [17] In this study, no significant difference between the healthy eyes and glaucoma eyes for the two major outcome measures was expected. In addition, the two glaucoma patients were in an early stage of disease and the IOP difference between the paired eyes were 1 and 2 mmHg, respectively.

In conclusion, this study shows that the CLS outputs reflect known changes in IOP that are in agreement with pneumatonometry measurements of the contralateral eye. This agreement was assessed both for the characteristic IOP rise when individuals transit from the wake to the sleep state (W/S slope) as well as for the overall 24-h curve. Furthermore, we demonstrate that the CLS detects short-term changes of IOP related to the cardiac cycle with good accuracy. These data provide additional information to support the use of the CLS as an ambulatory tool for 24-h assessment of IOP-related patterns.

## Supporting Information

S1 CONSORT Checklist CONSORT checklist. (PDF)

S1 Protocol Trial Protocol. (DOCX)
