# Supplementary material for: Efficacy of a Contact Lens Sensor for Monitoring 24-H Intraocular Pressure Related Patterns
Source: PLoS One. 2015 May 5;10(5):e0125530. doi: 10.1371/journal.pone.0125530 (PMC4420265; doi:10.1371/journal.pone.0125530)
Supplement: S1 Protocol — (DOCX) [file pone.0125530.s002.docx]

**CLINICAL INVESTIGATION PLAN**

**efficacy of 24-hour intraocular pressure fluctuation recording with the sensimed Triggerfish^®^ contact lens sensor**

Study number 10/09

March 4^th^, 2011

Protocol approved by:

Principal Investigator and Sponsor:

I have reviewed this clinical investigation plan and approve the conduct of this study.

John Liu, PhD Date

Sensimed:

I have reviewed this clinical investigation plan and approve the conduct of this study.

René Goedkoop, MD, CMO, Sensimed AG Date

Statistician:

I have reviewed this clinical investigation plan and approve the conduct of this study.

Yossi Tal, PhD, biostatistician, President, TechnoSTAT Date

This protocol contains confidential information and is intended solely for the guidance of the clinical investigation. This protocol may not be disclosed to parties not associated with the clinical investigation or used for any purpose without the prior written consent of the LKP and/ or of the sponsor (if applicable).

Principal Investigator

John Liu, PhD

Professor of Ophthalmology

University of California San Diego

Hamilton Glaucoma Center

9500 Gilman Drive

La Jolla, CA 92093-0946

USA

Phone: +1(858) 534-7056

Fax : +1(858)534-7014

Email: joliu@ucsd.edu

Sensimed

René Goedkoop, MD

Sensimed AG

Route de Chavannes 37

CH-1007 Lausanne

Switzerland

Phone: +41 21 621 9191

Fax: +41 21 621 9193

Email: rene_goedkoop@sensimed.ch

Statistician

Yossi Tal, PhD

TechnoSTAT

34^th^ Jerusalem St.

Beit Gamla, 5^th^ floor

Raanana, 43501

Israel

Trial Coordinator (Sensimed)

Jeanette Lindell

Sensimed AG

Route de Chavannes 37

CH-1007 Lausanne

Switzerland

Phone: +41 21 621 9191

Fax: +41 21 621 9193

Email: jeanette_lindell@sensimed.ch

Monitor

Name

Company

Address 1

Address 2

Country

Phone:

Fax:

Email:

Protocol synopsis

| Title | Efficacy of 24-hour intraocular pressure fluctuation recording with the SENSIMED Triggerfish^®^ contact lens sensor |
| --- | --- |
| Indication | Primary open-angle glaucoma patients and healthy subjects |
| Objectives | Safety and effectiveness of the SENSIMED Triggerfish^®^ (TF) device in continuous recording of relative fluctuation in intraocular pressure (IOP). Safety will be assessed by recording of adverse events (AEs) during 24 hours of continuous TF monitoring. TF efficacy will be evaluated by demonstrating TF ability to detect:   1. The known phenomenon of increase in IOP when moving from waking state to going to bed, as reflected in pneumotonometer measurements 2. Ocular Pulse frequency relative to direct measurement of Heart Rate (HR). |
| Trial Design | Single-center, open label, exploratory investigation |
| Inclusion Criteria | - Signed informed consent for the investigation - Diagnosis of primary open angle glaucoma (POAG), including normal tension glaucoma, or healthy subjects, including subjects with ocular hypertension for whom no evidence or suspicion of structural or functional glaucomatous damage exists - No anti-glaucomatous drug treatment or washed-out for 4 weeks - IOP symmetry of +/- 3 mmHg between fellow eyes - Age 18–80 years - Not more than 4 diopters spherical equivalent on both eyes - Not more than 2 diopters cylinder equivalent on both eyes - For women of childbearing potential, adequate contraception |
| Exclusion Criteria | - Patients who have had ocular surgery within the last 3 months. - Corneal or conjunctival abnormality hindering SENSIMED Triggerfish^®^ Sensor adaptation - Wear of full frame metallic glasses during SENSIMED Triggerfish^®^ monitoring - Severe dry eye - Secondary forms of open angle glaucoma (OAG) - Allergy to corneal anesthetic - Patients with contraindications for silicone contact lens wear - Pregnancy and lactation - Patients not able to understand the character and individual consequences of the investigation - Simultaneous participation in other clinical research |
| Medical Device | System consisting of: SENSIMED Triggerfish^®^ Ocular Telemetry Sensor, Data Cable, Antenna, Recorder and Software |
| Sample Size | 30 eyes of 30 patients showing at least 3 mmHg difference in IOP between before and after going to bed |
| Statistical Analysis | 6 weeks after End of Study (last patient off study) |
| Inclusion period | 6 months |

Abbreviations

AE adverse event

BCVA best corrected visual acuity

CCT central corneal thickness

CE conformité européenne

CRF case report form

EDTRS early diabetes treatment retinopathy study

EOI end of investigation

FDA Food and Drug Administration

GAT Goldmann applanation tonometry

IOP intraocular pressure

IRB institutional review board

ISO International Standard Organisation

LPLV last patient last visit

mmHg millimetre of mercury

TBUT tear breakup time

SDV source data verification

UCSD University of California, San Diego

USB universal serial bus

VAS visual analogue scale

TABLE OF CONTENTS

[Protocol synopsis 3](#_Toc278885192)

[Abbreviations 5](#_Toc278885193)

[TABLE OF CONTENTS 6](#_Toc278885194)

[PART A. purpose 11](#_Toc278885195)

[1 objectives and hypotheses 11](#_Toc278885196)

[1.1 Name of investigational device 11](#_Toc278885197)

[1.2 Proposed intended use of the device 11](#_Toc278885198)

[1.3 Objectives 11](#_Toc278885199)

[1.4 Duration 11](#_Toc278885200)

[PART B. protocol 12](#_Toc278885201)

[2 Introduction 12](#_Toc278885202)

[3 Preclinical testing of SENSIMED Triggerfish 13](#_Toc278885203)

[3.1 In vitro 13](#_Toc278885204)

[3.2 Ex vivo 13](#_Toc278885205)

[3.2.1 Wired model 13](#_Toc278885206)

[3.2.2 Wireless model (Ocular Telemetry Sensor) 13](#_Toc278885207)

[3.2.3 In vivo 13](#_Toc278885208)

[4 Previous clinical experience 14](#_Toc278885209)

[4.1 Wired prototype 14](#_Toc278885210)

[4.1.1 Changing position 14](#_Toc278885211)

[4.1.2 Anesthesia 14](#_Toc278885212)

[4.1.3 Eyes open without blinking 14](#_Toc278885213)

[4.1.4 Valsalva maneuver 14](#_Toc278885214)

[4.2 Wireless prototype 15](#_Toc278885215)

[4.2.1 Wearing comfort in healthy volunteers during 24-hour wear in ambulatory mode – investigation 09/01 15](#_Toc278885216)

[4.2.2 Safety and tolerability in healthy volunteers and glaucoma patients during 24-hour continuous IOP recording – investigation 09/02 18](#_Toc278885217)

[5 Design of the clinical investigation 19](#_Toc278885218)

[5.1 Title of the investigation 19](#_Toc278885219)

[5.2 General 19](#_Toc278885220)

[5.2.1 Safety endpoints 19](#_Toc278885221)

[5.2.2 Primary efficacy endpoints 19](#_Toc278885222)

[5.2.3 Secondary efficacy endpoints 20](#_Toc278885223)

[5.2.4 Performance endpoint 20](#_Toc278885224)

[5.2.5 Equipment and methods 20](#_Toc278885225)

[5.2.6 Device exposure 20](#_Toc278885226)

[6 Subjects 20](#_Toc278885227)

[6.1 Patient withdrawal and replacement 21](#_Toc278885228)

[6.2 In all cases of patient withdrawal, the patient will receive a final ophthalmological examination as stated in 7.4. Inclusion / exclusion criteria 21](#_Toc278885229)

[6.2.1 Inclusion criteria 21](#_Toc278885230)

[6.2.2 Exclusion criteria 21](#_Toc278885231)

[7 Procedures 22](#_Toc278885232)

[7.1 Baseline visit 22](#_Toc278885233)

[7.2 Circadian synchronization 23](#_Toc278885234)

[7.3 Day 1 23](#_Toc278885235)

[7.4 Day 2 24](#_Toc278885236)

[7.4.1 Ocular pulse assessment 25](#_Toc278885237)

[7.4.2 Final procedures 25](#_Toc278885238)

[7.5 Procedures to assess safety 25](#_Toc278885239)

[7.6 In the current investigation safety is assessed through the collection of adverse events and serious adverse events (cf section 8).Procedures to assess efficacy 25](#_Toc278885240)

[7.7 Procedures to assess performance 26](#_Toc278885241)

[7.8 Procedures carried out by Sensimed representatives 26](#_Toc278885242)

[7.9 Concomitant medications / treatments / conditions 26](#_Toc278885243)

[7.9.2 Concomitant Treatments 26](#_Toc278885244)

[7.10 Duration of follow-up 26](#_Toc278885245)

[8 Adverse events, adverse device effects and non-medical complaints 26](#_Toc278885246)

[8.1 Adverse Event Definitions 26](#_Toc278885247)

[8.2 Complaint definition 27](#_Toc278885248)

[8.3 Eliciting Adverse Event Information 28](#_Toc278885249)

[8.4 Recording and Reporting of Adverse Events 28](#_Toc278885250)

[8.4.1 Reporting of Serious Adverse Events (SAE), Serious Adverse Device Effects (SADE) and Unanticipated Serious Adverse Device Effects (UADE) 28](#_Toc278885251)

[8.4.2 Reporting of Adverse Device Effects (ADE) 28](#_Toc278885252)

[8.5 Abnormal test findings 28](#_Toc278885253)

[8.6 Causality and severity assessment 29](#_Toc278885254)

[8.7 Reporting Period 30](#_Toc278885255)

[9 Statistical section 30](#_Toc278885256)

[9.1 Study Objectives 30](#_Toc278885257)

[9.2 Study Design 30](#_Toc278885258)

[9.3 Population 31](#_Toc278885259)

[9.4 Overview of Investigation 31](#_Toc278885260)

[9.4.1 IOP Change from Wake to Sleep States 31](#_Toc278885261)

[9.4.2 Ocular Pulse 32](#_Toc278885262)

[9.5 Endpoints 32](#_Toc278885263)

[9.5.1 Safety 32](#_Toc278885264)

[9.5.2 Primary Efficacy 32](#_Toc278885265)

[9.5.3 Secondary Efficacy 33](#_Toc278885266)

[9.5.4 Performance 33](#_Toc278885267)

[9.6 Analysis Sets 33](#_Toc278885268)

[9.6.1 Safety Analysis Set 33](#_Toc278885269)

[9.6.2 Primary Efficacy Analysis Set 33](#_Toc278885270)

[9.6.3 Performance Analysis Set 34](#_Toc278885271)

[9.7 Sample Size Considerations 34](#_Toc278885272)

[9.8 Statistical Analysis 35](#_Toc278885273)

[9.8.1 Overview 35](#_Toc278885274)

[9.8.2 Subject Disposition 35](#_Toc278885275)

[9.8.3 Safety 35](#_Toc278885276)

[9.8.4 Primary Efficacy 35](#_Toc278885277)

[9.8.5 Secondary Efficacy 37](#_Toc278885278)

[9.8.6 Performance 37](#_Toc278885279)

[9.9 Interim Analysis 37](#_Toc278885280)

[10 scientific analysis of the protocol 38](#_Toc278885281)

[11 Administrative and legal aspects 39](#_Toc278885282)

[11.1 Approval of Trial Protocol and Amendments 39](#_Toc278885283)

[11.2 Continuous Information to competent Ethics Committee 39](#_Toc278885284)

[11.3 Submission to Regulatory Authorities 39](#_Toc278885285)

[11.4 Quality assurance 39](#_Toc278885286)

[11.5 Informed consent 39](#_Toc278885287)

[11.6 Insurance 40](#_Toc278885288)

[11.7 Responsibilities of Investigator 40](#_Toc278885289)

[11.8 Data Management 40](#_Toc278885290)

[11.9 Confidentiality 41](#_Toc278885291)

[11.10 Early termination or Suspension of the Investigation 41](#_Toc278885292)

[11.11 End of the Investigation and Final Report 41](#_Toc278885293)

[11.12 Publication Policy 41](#_Toc278885294)

[PART C. risk analysis 42](#_Toc278885295)

[12 risk analysis 42](#_Toc278885296)

[12.1 Anticipated clinical benefits 42](#_Toc278885297)

[12.2 Anticipated risks 42](#_Toc278885298)

[12.2.1 SENSIMED Triggerfish^®^ 42](#_Toc278885299)

[12.2.2 Tonometer 43](#_Toc278885300)

[12.3 Benefit risk ratio 43](#_Toc278885301)

[12.4 Description of patient population 43](#_Toc278885302)

[PART D. Device description 44](#_Toc278885303)

[13 device description 44](#_Toc278885304)

[13.1 General 44](#_Toc278885305)

[13.1.1 SENSIMED Triggerfish^®^ Ocular Telemetry Sensor 44](#_Toc278885306)

[13.1.2 SENSIMED Triggerfish^®^ Antenna 45](#_Toc278885307)

[13.1.3 SENSIMED Triggerfish^®^ Recorder 45](#_Toc278885308)

[13.1.4 SENSIMED Triggerfish^®^ Data Cable 45](#_Toc278885309)

[13.1.5 SENSIMED Triggerfish^®^ Software 45](#_Toc278885310)

[13.2 Therapeutic/diagnostic Effects 45](#_Toc278885311)

[13.3 Device operation 45](#_Toc278885312)

[13.4 Manufacturer 46](#_Toc278885313)

[13.5 Catalogue information 46](#_Toc278885314)

[13.6 Device traceability 46](#_Toc278885315)

[PART E. investigation monitoring 47](#_Toc278885316)

[14 monitoring procedures 47](#_Toc278885317)

[PART F. labeling 48](#_Toc278885318)

[PART G. consent materials 49](#_Toc278885319)

[PART H. irb/EC information 50](#_Toc278885320)

[PART I. other institutions 51](#_Toc278885321)

[PART J. additional records and reports 52](#_Toc278885322)

[PART K. Literature 53](#_Toc278885323)

[PART L. anticipated adverse events 55](#_Toc278885324)

[15 adverse device effects and complications 55](#_Toc278885325)

[15.1 Related to SENSIMED Triggerfish^®^ Sensor 55](#_Toc278885326)

[15.2 Related to SENSIMED Triggerfish^®^ Antenna 57](#_Toc278885327)

[15.3 Related to SENSIMED Triggerfish^®^ Recorder 58](#_Toc278885328)

[15.4 Related to the Goldmann applanation tonometer and pneumatonometer 58](#_Toc278885329)

[15.5 Related to proparacaine 59](#_Toc278885330)

[PART M. investigator endorsement page 60](#_Toc278885331)

[PART N. study overview – schematic 61](#_Toc278885332)

1. purpose

# objectives and hypotheses

## Name of investigational device

SENSIMED Triggerfish^®^ System, consisting of Sensor, Data Cable, Antenna, Recorder and Software.

## Proposed intended use of the device

The SENSIMED Triggerfish^®^ is intended as a tool for the clinician to continuously record the timing of relative changes in IOP for up to 24 hours.

The SENSIMED Triggerfish^®^ is intended for prescription-use only.

SENSIMED Triggerfish^®^ Ocular Telemetry Sensor **is not intended for vision correction**.

## Objectives

The objectives of this study are to assess the safety and efficacyof the SENSIMED Triggerfish^®^ (TF) device in continuous recording of relative fluctuation in intraocular pressure (IOP). Safety will be assessed by recording of adverse events (AEs) during the 24 hours of continuous TF monitoring. TF efficacy will be evaluated by demonstrating TF ability to detect:

1. The known phenomenon of increase in IOP when moving from wake state to going to bed, as reflected in pneumotonometer measurements
2. Ocular Pulse frequency relative to direct measurement of Heart Rate (HR).

## Duration

The expected study duration is 6 months.

1. protocol

# Introduction

Although researchers and clinicians recognize the need for continuous IOP fluctuation recording of patients with glaucoma or ocular hypertension, no method is yet available. IOP fluctuation recording would give a great improvement in managing glaucoma and in testing drugs that could lower intraocular pressure (IOP). The Sensimed Triggerfish is a novel and minimally invasive approach to IOP fluctuation recording. The key element of this measurement method is a soft contact lens with an embedded micro fabricated strain gauge allowing the measurement of changes in corneal curvature correlated to variations in IOP, regardless the patient’s position and activity. A prototype of this Triggerfish was adapted and tested on enucleated porcine eyes. To verify the measurement principle of the device, the posterior chamber of the pig eyes was cannulated and connected to a syringe pump and a pressure sensor for precise control of IOP. The measurements of the contact lens were then compared to the ones from the pressure sensor, while pressure variations were induced through the cannula. The Triggerfish shows the potential for continuously recording IOP fluctuation in enucleated porcine eyes, in all cases the Triggerfish followed the IOP variations very well. The device is placed in the same way as a corrective contact lens, no anaesthesia is required, and vision remains almost unimpaired. A small first in human 24-hour study with healthy subject was performed in Lausanne, Switzerland and showed good tolerability in all participating subjects. The device was authorized for the European market in February 2009. A second study was performed in Switzerland on 10 healthy subjects in 2009 and showed good tolerability over 24 hours. Another study in Germany recruited 40 subjects for 24-hour recording and showed good safety and tolerability.

Goldmann applanation tonometry (GAT) has been the gold standard for measuring IOP in the last 50 years, it is widely established. Factors that may affect GAT readings are corneal thickness, corneal curvature, structural corneal rigidity and astigmatism. Recent studies showed that GAT may overestimate IOP in eyes with thicker corneas and underestimate IOP in thinner corneas. Corneal pachymetry should be performed when assessing IOP using GAT. Its weaknesses include a hygienic problem caused by the necessity for head of the tonometer to be in direct contact with the anesthetized cornea. Furthermore, the patient’s collaboration is required, the patient has to visit the ophthalmologist’s office, and during measurements, some aqueous humor may be displaced.

Most importantly, so far IOP fluctuation can’t be recorded continuously. This leaves doubt as to control IOP over 24 hours and especially at night. Continuous IOP measurement would, thus, lead to further understanding of normal IOP variations as well as improve treatment monitoring.

# Preclinical testing of SENSIMED Triggerfish

## In vitro

The SENSIMED Triggerfish^®^ has been shown to conform to a number of international standards. For practical purposes, these are not listed here.

## Ex vivo

### Wired model

The functionality of a wired version of the Sensor was evaluated in enucleated and pressure-controlled pig eyes. The eyes were cannulated in the posterior chamber and connected to a bag with saline and to a syringe pump. A pressure Sensor was placed close to the eye. Six eyes were stimulated by applying ramps of increasing and decreasing IOP. In all cases, the Sensor signal followed the IOP variations very well.

### Wireless model (Ocular Telemetry Sensor)

The telemetric contact lens Sensor functionality was evaluated on enucleated, juvenile pig eyes. The eyes were cannulated in the anterior chamber and connected to a column filled with saline solution. The amount of saline was controlled by a syringe pump and varied to create different pressures in the enucleated eyes as controlled by a manometer placed at the same height as the eye. Ten eyes were used. Since Sensor fitting is not optimized for porcine eyes and since eye size varied significantly from one eye to the other, each eye was calibrated separately at two known pressures to set the correspondence between IOP and the manometric pressure.

Two experiments were carried out. In the first, IOP was varied dynamically between 11 and 14 mmHg at a frequency simulating ocular pulsation. The Sensor followed the variation in IOP very well. There was a time delay between the Sensor signal and the pressure reading, most probably due to the fluidic resistance of saline moving in the liquid circuit. In the second experiment, the IOP of the enucleated pig eyes was varied in static steps of 1 mmHg between 20 and 30 mmHg to test the repeatability and linearity of the wireless recording. The linear regression coefficient obtained was very high (0.9935) and the 95% confidence interval for repeatability was ± 0.2 mmHg (values for one eye).

### In vivo

No non-clinical in vivo testing has been done.

# Previous clinical experience

## Wired prototype

A wired prototype of the Sensor was evaluated qualitatively for functionality on seven healthy volunteers. The study, in accordance with ICH/GCP, was approved by the IRB and informed consent was obtained from the patients prior to any study-related procedures.

The signal was recorded for the patient staying in sitting, lying and standing position, each maintained for 5 to 10 minutes. Finally the patient sat on a chair and was asked to do the Valsalva maneuver three times. Only right eyes were tested. One drop of oxybuprocaine was instilled in the right eye prior to the placing of the Sensor. A new drop of anesthesia was applied if requested by the patient during the procedure. The protocol was executed twice, once with the eye open and once with a plaster on the right eye.

IOP was assessed using a Goldmann applanation tonometer and a TonoPen, also of applanation type but capable of assessing IOP in the lying position, though less precise. All IOP assessments were made in the left eye while the Sensor was worn on the right eye.

There was no correlation between the quality of the signals recorded with the Sensor and the biometric data of the subjects. Various signals of interest were recorded during the trial, in particular a similar signal was recorded in all patients when they were asked to stop blinking for as long as they could. A signal that can be interpreted as the ocular pulsation was seen on some patients when the recording was performed with the eyelid closed. This signal correlated with the heart rate of the subjects.

### Changing position

For most patients, the changes in IOP measured on the left eye with the TonoPen after changing position were small. The signal recorded with the Sensor was also stable. However, in two patients changes of the baseline were observed and seemed correlated with the change in body position. No major difference was noted when this test was carried out with the right eye closed by a sterile gauze, except that the noise level was slightly lower.

### Anesthesia

No effect was seen on the recorded signal when a drop of oxybuprocaine was instilled in the right eye while wearing the Sensor.

### Eyes open without blinking

A similar signal was obtained in all patients who carried out the test. The signal first decreased, and later increased to reach an asymptote.

### Valsalva maneuver

Specific signals were recorded when subjects performed the Valsalva maneuver. More interindividual variation was observed during this maneuver than during the open eye test. However, the signal recorded during the Valsalva maneuver was clearly distinguishable from that recorded with open eyes without blinking. The signal recording during Valsalva maneuver with closed eye was more variable than with open eye.

## Wireless prototype

### Wearing comfort in healthy volunteers during 24-hour wear in ambulatory mode – investigation 09/01

This investigation evaluated the level of comfort and the safety of extended 24-hour SENSIMED Triggerfish^®^ Sensor wear during normal activities of 10 healthy volunteers.

Subjects were 24 years or older. None of the subjects had concomitant disease at the time of the study or any previous ocular surgery. Mean Goldmann applanation tonometry (GAT) IOP was 14.70 ± 3.30 mmHg (range 10.0-20.0 mmHg) in both eyes at baseline and 13.70 ± 2.41 mmHg (range 10.0 to 18.0 mmHg) in the study eye. This difference was not statistically significant. Furthermore, all subjects had a normal cornea according to the investigator’s grading (observation of corneal morphological state and cornea-Sensor interface). The grading changed in a total of 5 subjects from a score of 1 (normal) to 1 (insignificant state; 1 subject) and 2 (significant state; 4 subjects). This change was observed as early as 5 minutes after Sensor installation (3 subjects, grade 1) with new cases after 4 hours (5 subjects, grade 1). Grade 2 scores were reported after Sensor removal only. Conjunctival injection appeared in one subject 5 minutes after Sensor installation and was present in 5 subjects after 24 hours. One more subject presented pericorneal injection after 24 hours. Tear breakup time (TBUT) at baseline was reduced in the majority of subjects (mean 6.1 seconds, median 6.0 seconds) indicating reduced tear film quality without visible corneal signs of dry eye disease. The height of the tear film meniscus was normal to high in 9 of 10 subjects, indicating that the aqueous layer of the tear film was normal while the non-aqeuous layer appeared to be reduced in the study population. Wetability, defined as pre-Sensor tear film quality, was characterized as good in 9 of 10 subjects after 5 minutes and remained stable throughout the observation period, which is in line with the observed height of the tear film meniscus.

Mean device exposure time was 23.23 ± 0.40 hours (range 22.90 to 24.08 hours). To be included in the primary analysis set, subjects’ device exposure had to be at least 23 hours. Two subjects wore the device for slightly less than 23 hours, however, given their complete sets of data and the clinical irrelevance associated with the small difference in device exposure observed, it was decided to retain these two subjects for the primary analysis. The results were similar with and without these two subjects.

In this study, the Sensor was well tolerated over a 24-hour period. The mean subjective comfort score was overall high between 7.35 and 8.35 (out of 10) without statistically significant fluctuations over time.

Two subjects presented pingueculae in the study eye. In one subject, the pinguecula was located in the temporal quadrant of the eye and in the second there were nasal and temporal pingueculae. The former subject presented good centering of the Sensor throughout the 24 hours, while the latter presented an infero-nasally deviated Sensor. In the other subjects, there was horizontal deviation in 4 out of 8 and vertical deviation in 5 out of 8 subjects. Sensor centering did not seem to be influenced by the presence of pingueculae.

The nature of the Sensor movement was characterized as being fluid in 60 % of subjects 5 minutes after installation. The remainder showed no or limited Sensor movement. After 24 hours, there was no spontaneous Sensor movement in 90% of subjects. The same trend was observed for the push-up test (70% fluid after 5 minutes compared to 100% difficult after 24 hours). This is additionally underlined by the observation of the relative stability in the position of the telemetric microprocessor. On average, the difference in chip position between baseline and 24 hours corresponded to 1 clock hour of rotation. However, due to the statistical method used to calculate the rotation, the direction of rotation was not taken into account. The stable Sensor position could be responsible for the minor corneal surface irregularities seen after 24-hour wear. On the other hand, minimal horizontal and vertical Sensor displacement during recording can be assumed to be advantageous to signal consistency.

Another relevant corneal parameter in association with SENSIMED Triggerfish^®^ wear is central corneal thickness (CCT). This parameter changed from a mean of 549.40 μm (range 517.00 - 610.00 μm) to 549.10 (range 489.00 – 613.00 μm), which was not statistically significant. The fact that the CCT did not vary significantly before and after device application indicates that no major corneal oedema or metabolic suffering occurred during the 24-hour wear. A CCT difference of more than 20 µm, equal to the normally observed diurnal change in CCT, was used to classify subjects for either normal or abnormal CCT fluctuation over 24 hours. Two subjects showed significantly reduced CCT with differences of 25 and 28 μm after 24-hour Sensor wear respectively. At present, we do not have an explanation for the observed reduction in CCT in these subjects. Further investigation of this observation and its consequences is warranted.

The device had an effect on subjective and objective refraction. This effect was expected since the Sensor has a known residual optic power of approximately 1 dpt (data on file). The subjective refraction baseline mean spherical equivalent was -0.88± 1.93 dpt in the study eye. This changed to -0.18 ± 1.56 dpt. (5 min after device installation), 0.13 ± 1.94 (4 hours), 0.10 ± 2.18 (7 hours) and 0.10 ± 2.26 (24 hours). After device removal, mean spherical equivalent was -1.90 ± 1.88 dpt. The observed difference in the refraction spherical equivalent between baseline and Sensor removal was -1.03 ± 1.08 (range -3 to 0.75 dpt.). The difference at 24 hours and immediately after Sensor removal was -2.01 dpt. (p=0.0018). This difference was higher than that observed between baseline and immediately after Sensor installation, indicating that the Sensor induces a temporary myopisation. The subjective refraction cylinder equivalent changed from -0.55 ± 0.50 dpt. at baseline to -0.83 ± 0.49 dpt. after Sensor removal. This change was not statistically significant. The subjective refraction axis changed from 53.00 ± 56.63 ° at baseline to 42.50 ± 48.38 ° after Sensor removal. This change was statistically significant (p=0.004). . The Objective refraction carried out after Sensor removal confirmed this effect: The objective refraction baseline mean spherical equivalent was -0.93± 1.90 dpt in the study eye, this changed to -2.00± 1.97 dpt. after lens removal. This change was statistically significant (p=0.004). The objective refraction cylinder equivalent changed from -0.50 ± 0.41 dpt. at baseline to -0.73 ± 0.42 dpt. after Sensor removal. This change was not statistically significant. The objective refraction axis changed from 66.00 ± 65.69 ° at baseline to 75.50 ± 64.74 ° after Sensor removal. This change was statistically significant (p=0.002). Abnormal refraction difference over 24 hours was defined as more than 0.5 diopters for the objective and subjective refraction spherical and cylinder equivalent and more than 10° for the objective and subjective refraction axis. Eight of ten subjects exhibited abnormal refraction subjective spherical equivalent change, three of ten showed abnormal refraction subjective cylinder equivalent change and eight of ten subjects showed abnormal subjective refraction axis change. Nine of ten subjects exhibited abnormal refraction objective spherical equivalent change, three of ten showed abnormal refraction objective cylinder equivalent change and all ten subjects showed abnormal objective refraction axis change.

Baseline best-corrected visual acuity (BCVA) was 1.07 ± 0.18 (range 0.65 to 1.20). This changed to 0.56 ± 0.21 (5 minutes), 0.64 ± 0.22 (4 hours), 0.61 ± 0.26 (7 hours) and 0.70 ± 0.22 (24 hours). Immediately after Sensor removal, BCVA improved to 0.85 ± 0-26 (range 0.30 to 1.20). Best corrected visual acuity was reduced during Sensor wear and this reduction remained statistically significant immediately after Sensor removal. This can be explained by the Sensor inducing corneal surface irregularities, as measured by subjective refraction and by the known residual optic power of the Sensor (see above). A reduction in non-corrected visual acuity was observed but was not statistically significant (0.51 ± 0.30 at baseline to 0.34 ± 0.16 after Sensor removal, p 0.188). Abnormal BCVA change over 24 hours was defined as a loss of 0.3 or more. Two subjects exhibited abnormal BCVA difference.

Keratometry showed a statistically significant decrease in vertical corneal radius from baseline (7.53 ± 0.21 mm, range 7.18 to 7.88 mm) to immediately following Sensor removal (7.41 ± 0.21 mm, range 7.01 to 7.63 mm). Horizontal corneal radius changed from 7.65 (SD 0.27 mm, range 7.19 to 7.64 mm) to 7.59 (SD 0.33mm, range 7.20 to 7.55 mm) mm after 24 hours. All but one subject showed a reduction in vertical and horizontal corneal radius during the course of the investigation. Abnormal corneal radius change, vertical and horizontal, was defined as more than 0.1 mm. Five of ten subjects exhibited abnormal horizontal and six of ten showed abnormal vertical radius change after 24 hours.

Three adverse device effects (ADE) were observed. No subject presented more than one adverse device effect. All reported ADEs were microscopic scratches of the cornea. In accordance with the investigator’s common practice, prophylactic topical antibiotic treatment and lubricating eye drops were given. All ADEs resolved rapidly. No correlation was observed between the observed ADEs and the height of the tear film meniscus. One of the subjects had a significantly reduced TBUT (2 seconds) while the other two were at the limit of normality.

In summary, this 24-hour investigation confirmed the functionality and tolerability of SENSIMED Triggerfish^®^ in human volunteers.

### Safety and tolerability in healthy volunteers and glaucoma patients during 24-hour continuous IOP recording – investigation 09/02

The primary endpoint in this trial was level of discomfort associated with SENSIMED Triggerfish^®^ in the installed eye at 24 hours, measured by a Visual Analog Scale (VAS from 0 to 100, 0 meaning no discomfort, 100 meaning intolerable). Two populations were included using a 1:1 ratio: Subjects with glaucoma and a similar group of healthy subjects.

All subjects underwent an ophthalmological examination including objective and subjective refraction tests, best corrected visual acuity, topography, pachymetry, slit lamp examination and Goldmann tonometry, before and after SENSIMED Triggerfish^®^ recording. Slit lamp findings (lid erythema, lid edema, conjunctiva erythema, conjunctiva edema, corneal staining and intraocular inflammation) were graded according to scales. Visual acuity was assessed using early diabetic treatment retinopathy study (EDTRS) charts.

AEs for slit lamp findings were recorded whenever a progression of 2 grades or more over 24 hours was observed. Visual acuity reduction was recorded as an AE whenever a reduction of 15 letters (3 lines) or more was observed.

All but 2 of the 40 subjects completed the full 24 hours of SENSIMED Triggerfish^®^ wearing. One glaucoma subject dropped out due to improper device fitting and one healthy subject dropped out due to device being too big.

No significant baseline differences, for gender, age or GAT- IOP were found between the two study groups.

The mean time of SENSIMED Triggerfish^®^ wearing was 23 hours for both study groups.

There were no SAE’s reported, but there were some device related AE’s:

85% of the glaucoma subjects (17/20) and 75% of the healthy subjects (15/20) experienced an adverse event that was probably or definitely device related to study device. Only a small portion of these device related adverse events were deemed severe (20% for glaucoma and 5% for healthy subjects).

There were no significant differences between healthy and glaucoma subjects on VAS when tested using an independent groups Wilcoxon rank sum test with α = 0.10 (p = 0.41), so that the two groups were combined for describing VAS. Median VAS (combined group) was 19, with 53% of subjects at 20 and below. The mean VAS score was 24.33.

The secondary endpoints included ophthalmologic, slit lamp and GAT-IOP examination of the study eye.

Significant changes from baseline to after SENSIMED Triggerfish^®^ recording were found for the following ophthalmic parameters: corneal topography horizontal, objective refraction – cyl, objective refraction – sph, objective refraction – degree, subjective refraction – cyl, subjective refraction – sph, subjective refraction – degree and visual acuity; and for the following slit lamp parameters: conjunctiva edema, conjunctiva erythema, epithelial defects and lid edema. There was no significant change from baseline in GAT-IOP.

In conclusion the two study groups (glaucoma and healthy Subjects) may be combined in terms of assessing SENSIMED Triggerfish^®^ tolerability.

SENSIMED Triggerfish^®^ was well tolerated over the 24-hour study period and caused no Serious Adverse Events (SAE’s).

# Design of the clinical investigation

## Title of the investigation

Efficacy of 24-hour intraocular pressure fluctuation recording with the SENSIMED Triggerfish^®^ contact lens sensor

## General

This is a prospective, open-label cohort investigation.

### Safety endpoints

Adverse events (AE's) including serious AE's (SAE's), including severity, occurring at any time during the trial or follow up, whether or not deemed related to study device. Safety measurements will include standard slit-lamp examination.

### Primary efficacy endpoints

This study has the following two, co-primary TF endpoints for each subject:

1. Linear correlation between Time and Continuous TF recordings from approximately 4 hours preceding going to bed to 4 hours after. Relationship between TF recordings and Time will be obtained by modelling the former against the latter using a second order polynomial^[[1]](#footnote-1)^. Linear correlation between TF recordings and Time is the standardized β obtained from the model.
2. Percent difference between frequency of ocular pulsation and HR scored dichotomously.

### Secondary efficacy endpoints

Secondary efficacy endpoints include, for each subject:

- Subject Acceptance of TF, which will be measured on a 5 point visual analog scale (VAS) where 5, indicates high acceptance of TF and 1 indicates low acceptance.
- Categorization of subject diurnal rhythm of IOP as measured by TF and assignment to pre-specified diurnal rhythms identified from the literature.

### Performance endpoint

- Validity of TF recording at the interval and subject levels scored dichotomously:
  - 0 – invalid
  - 1 – valid

### Equipment and methods

Investigational data will be collected during patient visits and hospitalisation on site. All investigation related data will be recorded on CRFs. Delegated site personnel will be instructed to complete the CRFs after each visit. All investigational data will be analyzed 6 weeks after Last Patient Last Visit (LPLV).

All equipment used in this investigation is standard ophthalmology service equipment. Calibration and other equipment maintenance will be done at regular intervals as specified by site procedures. Equipment to be used in the study will be listed and maintained as a separate document. All tonometers used in this investigation will be calibrated.

IOP measurements will be assigned randomly to experienced researchers, adopting working schedule to avoid fatigue. Inter-operator IOP variability will be verified to be insignificant prior to the study. The same equipment (instruments) will be used for all patients.

Proparacaine will be used as corneal anaesthesia.

### Device exposure

The planned exposure time to SENSIMED Triggerfish is 24 hours ± 1 hour during one IOP fluctuation recording session. This includes 24-hour active IOP fluctuation recording and additional time necessary for the fitting control plus some variation due to logistic reasons.

# Subjects

The investigation will recruit male and female patients 18-80 years old and either diagnosed with primary open-angle glaucoma, including normal tension glaucoma or being healthy subjects including subjects with ocular hypertension.

30 eyes of 30 subjects showing at least 3 mmHg difference between wake and sleep IOP, as calculated as the mean of IOP during the 4 hours preceding and following going to bed, respectively, will be enrolled in the investigation.

Patients will be considered enrolled in the study upon signature of informed consent. A screening visit and one 24-hour IOP fluctuation recording session are planned for each patient. IOP fluctuation recording sessions will be carried out in a sleep unit. The expected duration of participation for each patient of 2 days over two to three weeks.

Patients will be recruited over a 6-month period. The total expected study duration is 6 months.

## Patient withdrawal and replacement

Patients will be immediately withdrawn from the study in the case of revoked consent or, in the investigator’s opinion, withdrawing the patient is in the patient’s best interest. If informed consent is revoked prior to device installation, the subject will be replaced.

If no Sensor (maximum two attempts with different Sensor sizes) can be adapted to the subject, no recording will be carried out and the Sensor removed from the eye of the subject. The subject will be withdrawn from the study and replaced.

Patients experiencing device-related Serious Adverse Events will be withdrawn from the study. With the consent of the patient, the 24-hour recording session will be repeated in case of device failure.

In all cases of patient withdrawal, the patient will receive a final ophthalmological examination as stated in 7.4. Inclusion / exclusion criteria.

### Inclusion criteria

Subjects must correspond to all other inclusion criteria in order to be eligible for the investigation.

- Signed informed consent for the investigation
- Diagnosis of primary open angle glaucoma (POAG), including normal tension glaucoma, or healthy subjects, including subjects with ocular hypertension for whom no evidence or suspicion of structural or functional glaucomatous damage exists
- No anti-glaucomatous drug treatment or washed-out for 4 weeks
- IOP symmetry of +/- 3 mmHg between fellow eyes
- Age 18–80 years
- Not more than 4 diopters spherical equivalent on both eyes
- Not more than 2 diopters cylinder equivalent on both eyes
- For women of childbearing potential, adequate contraception

### Exclusion criteria

Subjects presenting with any of the following criteria will not be included in the trial:

- Patients who have had ocular surgery within the last 3 months.
- Corneal or conjunctival abnormality hindering contact lens adaptation
- Wear of full frame metallic glasses during SENSIMED Triggerfish^®^ monitoring
- Severe dry eye
- Secondary forms of open angle glaucoma (OAG)
- Allergy to corneal anesthetic
- Patients with contraindications for silicone contact lens wear
- Pregnancy and lactation
- Patients not able to understand the character and individual consequences of the investigation
- Simultaneous participation in other clinical research

No patient will be allowed to participate in this trial more than once.

# Procedures

Patients accepting signing informed consent will receive screening procedures to determine their eligibility for the investigation. All eligible patients will be enrolled and receive IOP fluctuation recording during 24 hours. Left and right eyes will be recorded randomly.

## Baseline visit

Following the signature of informed consent, the procedures listed hereunder will be carried out on the study eye.

- Demographic data
- Medical and surgical history
- Concomitant medication and procedures
- Automated objective refraction test
- Best corrected visual acuity (BCVA)
- Slit lamp examination
- TBUT
- Corneoscleral profile evaluation
- Topography/keratometry
- Pachymetry
- Goldmann applanation tonometry (three readings) and pneumatonometry in each eye
- For women of childbearing potential, pregnancy test
- Inclusion and exclusion criteria

For GAT, one drop of proparacaine will be instilled in the eye prior to the measurement. The three readings will be taken within at one-minute intervals.

IOP will be measured by GAT and pneumatonometer in the sitting position in both eyes. Then, the subjects will lie down for at least 15 minutes and the IOP will be measured with the pneumatonometer in both eyes.

A baseline evaluation of the corneoscleral profile and topography/keratometry will be done to determine the appropriate Sensor base curve for the patient, according to the SENSIMED Triggerfish^®^ User Manual.

## Circadian synchronization

7 days prior to the IOP fluctuation recording, subjects will adopt a regular dark-sleep cycle close to 11 PM to 7 AM, with a maximum deviation of 1 hour. Subjects will be monitored for light exposure and physical activity. In addition, they will keep a sleep-wake diary.

All subjects will be instructed to abstain from alcohol for 3 days and caffeine for 1 day before the IOP fluctuation recording and not to wear contact lenses within the 24 hours preceding Day 1.

## Day 1

Subjects will enter the sleep unit at 2 PM ± 2 hours.

The following procedures will be carried out prior to IOP fluctuation recording:

- Best corrected visual acuity (BCVA)
- Slit lamp examination
- Pachymetry
- Goldmann applanation tonometry (GAT, three readings in each eye)
- Concomitant medication and procedures
- Adverse events and complaints

Prior to GAT, one drop of proparacaine will be instilled in the eye. The three tonometric measurements will be done at one-minute intervals.

The three reference GAT IOP measurements will be done in each eye immediately prior to the installation of the SENSIMED Triggerfish^®^ Sensor in one eye by the Investigator or delegated staff. Left and right eyes will be selected based on randomization.

If the Sensor base curve selected for the patient in line with the SENSIMED Triggerfish^®^ User Manual does not adapt appropriately to the patient’s eye, the initially selected Sensor may be removed and an attempt with a Sensor of different base curve done. A Sensor with the base curve expected to yield appropriate adaptation will be selected for the second attempt. The investigator will not attempt to install more than two Sensors to the same eye of a patient or healthy subject. The Sensor showing the better adaptation on the patient’s/healthy subject eye will be used for the recording. The other Sensor will be discarded. Any recording done with a Sensor having a base curve other than that indicated for the patient in the SENSIMED Triggerfish User Manual should be recorded, with motivation, in the patient’s CRF.

When the adaptation of the Sensor on the patient’s eye is satisfying, the recording will be initiated. The device will be used according to the manufacturer’s instructions.

Subjects will remain inside the sleep unit during the entire study. Light-dark cycles will be controlled and their onset will be individually adapted to match the subjects’ accustomed circadian cycle. Room activities will be taped. Patients will record their activities, environment and any particular events by the means of a multiple choice patient diary at the end of every 30-minute period throughout the recording.

If patients wish to read during the recording session, the reading material should be placed at eye level to assure communication between the Sensor and the Antenna. Full-frame metal glasses are prohibited throughout the recording since they are known to interfere with the device energy and data transfer.

During the IOP fluctuation recording, IOP measurements will be taken in the non-SENSIMED Triggerfish^®^ eye using a pneumatonometer, as follows:

- IOP (fellow eye) every two hours in the sitting position during the wake period
- 30 minutes after bedtime and then every 2 hours in the supine position

In the dark period, IOP measurements will be done in as near total darkness as possible (e.g. under red light intensity < 5 lux). Subjects will be awakened if necessary. At each time point and if needed, a reading will be taken after instillation of proparacaine within a few minutes.

## Day 2

IOP measurements in the fellow eye will be continued:

- Every two hours throughout the dark-sleep period, in supine position
- 30 minutes after waking, is sitting position and then every two hours until the full 24 hours

At each time point, measurements of IOP using the tonometer will be taken after instillation of proparacaine if needed. In the dark period, IOP measurements will be done in as near total darkness as possible. Subjects will be awakened if necessary. After an 8-hour dark-sleep period, lights will be turned on and subjects will be awakened, if needed.

### Ocular pulse assessment

At three times during the sleep period, event-related IOP fluctuation will be assessed. The following assessment will be done:

- Heart rate over 6 minutes

### Final procedures

After 24 hours the IOP fluctuation recording is completed and the device will be removed from the patient by the investigator or delegated staff. Recording data will be downloaded to the investigator’s computer. The following procedures will be carried out in both eyes:

- SENSIMED Triggerfish^®^ removal
- Best corrected visual acuity (BCVA)
- Slit lamp examination
- Pachymetry
- Goldmann applanation tonometry (GAT, three readings in each eye)
- Sleep quality assessment
- Visual analogue scale
- Collection of patient diary
- Concomitant medication and procedures
- Adverse events and complaints

## Procedures to assess safety

In the current investigation safety is assessed through the collection of adverse events and serious adverse events (cf section 8). Conjunctival redness and corneal staining will be graded according to Efron scales. Grades 0, 1 and 2 will be considered normal.

## Procedures to assess efficacy

SENSIMED Triggerfish^®^ efficacy is studied as the primary objective in this investigation. SENSIMED Triggerfish^®^ measures will be compared to parallel IOP measurements on fellow eye.

In addition, secondary efficacy will be investigated by subject acceptance, where subjects will be asked to indicate their acceptance of SENSIMED Triggerfish on a 5-point visual analog scale, where 5 indicates high acceptance and 1 indicates low acceptance.

Moreover, subjects’ diurnal rhythm as recorded with SENSIMED Triggerfish will be categorized according to pre-specified diurnal rhythms identified from literature.

## Procedures to assess performance

Performance is investigated by the dichotomous evaluation of the SENSIMED Triggerfish recording validity, where 0 means invalid recording and 1 valid recording in the current investigation. Non-medical complaints will be collected (cf section 8).

## Procedures carried out by Sensimed representatives

Training on the handling and operation of SENSIMED Triggerfish^®^ will be provided by Sensimed employees or representatives. Sensimed employees and representatives may assist to the installation/removal of SENSIMED Triggerfish^®^ on/from patients or healthy subjects upon agreement with the investigator and will provide technical support, remote or on site, on SENSIMED Triggerfish^®^ throughout the investigation.

## Concomitant medications / treatments / conditions

#### Prior and Concomitant medical conditions

Relevant prior and ongoing (at screening) concomitant medical conditions and procedures will be recorded on the Medical and Surgical history pages of the patient’s CRF. New illness and worsening of ongoing illness will be reported as AE, unless in line with expected disease progression.

### Concomitant Treatments

There are no prohibited treatments in this investigation. Any ongoing or new drug therapy will be recorded on the appropriate pages of the patient’s CRF from the date of informed consent.

## Duration of follow-up

In the case of an ophthalmic adverse event, patient should receive a follow-up ophthalmic examination in order to record the event outcome. Outside this procedure, there is no follow-up planned beyond the IOP fluctuation recording session in this investigation.

# Adverse events, adverse device effects and non-medical complaints

## Adverse Event Definitions

**Adverse event (AE):** Any untoward medical occurrence in a subject.

**Adverse device effect:** Any untoward and unintended response to a medical device.

**Life-threatening adverse event:** Any adverse event that places the subject, in view of the investigator-sponsor, at immediate risk of death from the event as it occurred (i.e., does not include an adverse event that, had it actually occurred in a more severe form, might have caused death).

**Serious adverse event (SAE):** Any adverse event that:

- Led to death
- Led to a serious deterioration in the health of the subject that
- Resulted in life-threatening illness or injury,
- Resulted in permanent impairment of a body structure or a body function ,
- Required in-patient hospitalization or prolongation of existing

hospitalization,

- Resulted in medical or surgical intervention to prevent permanent impairment to a body structure or a body function
- Led to foetal distress, foetal death or a congenital abnormality or birth defect

**Serious adverse device effect:** Adverse device effect that has resulted in any of the consequences characteristic of a serious adverse event or that might have led to any of these consequences if suitable action had not been taken or intervention had not been made or if circumstances had been less opportune.

*Hospitalization* shall include any initial admission (even if less than 24 hours) to a healthcare facility as a result of a precipitating clinical adverse event; to include transfer between hospital and intensive care unit. Hospitalization or prolongation of hospitalization in the absence of a precipitating, clinical adverse event (e.g. for a preexisting condition not associated with a new adverse event or with a worsening of the preexisting condition; admission for a protocol-specified procedure) is not, in itself, a serious adverse event.

**Unanticipated adverse device effect:** any serious adverse effect on health or safety or any life-threatening problem or death caused by, or associated with, a device, if that effect, problem, or death was not previously identified in nature, severity, or degree of incidence in the investigational plan or application (including a supplementary plan or application), or any other unanticipated serious problem associated with a device that relates to the rights, safety, or welfare of subjects.

## Complaint definition

**Complaint:** Written, electronic or oral communication that alleges deficiencies related to the identity, quality, durability, reliability, safety or performance of an investigational device. These can include complaints that might have led to a medical occurrence if a) suitable action had not been taken or b) intervention had not been made or c) if circumstances had been less fortunate.

NOTE: Complaints involving a medical occurrence are handled under the adverse event system (cf section 8.1;8.3-8.7).

## Eliciting Adverse Event Information

Clinical study patients will be routinely questioned about adverse events at study visits.

## Recording and Reporting of Adverse Events

All observed or volunteered adverse events (serious or not) and abnormal test findings, regardless of treatment group, if applicable, or suspected causal relationship to the investigational device or, if applicable, other study treatment or diagnostic product(s) will be recorded in the subjects’ CRF. For all adverse events, sufficient information will be pursued and/or obtained so as to permit 1) an adequate determination of the outcome of the effect (i.e. whether the event should be classified as a serious adverse event) and 2) an assessment of the causal relationship between the adverse event and the investigational device or, if applicable, the other study treatment or diagnostic product(s).

Adverse events felt to be associated with the investigational device will be followed until the event (or its sequelae) or the abnormal test finding resolves or stabilizes at a level acceptable to the investigator or until 30 days after the subject’s last exposure to the investigational device, whichever comes first.

### Reporting of Serious Adverse Events (SAE), Serious Adverse Device Effects (SADE) and Unanticipated Serious Adverse Device Effects (UADE)

All SAEs, SADEs and UADEs are to be immediately (without undue delay and no later than 24 hours) reported to Sensimed AG, using the AE Report Form, when gaining knowledge of the event:

SENSIMED AG

Email: AE@sensimed.ch

SADEs and UADEs are to be reported, by the investigator, to the competent Ethics Institutional Review Board (IRB), within 10 days of gaining knowledge of the event if there is a possible, probable or definite causal relationship with the investigational device.

### Reporting of Adverse Device Effects (ADE)

All ADEs are to be reported to Sensimed AG, using the appropriate form, within 7 days of gaining knowledge of the event, to:

SENSIMED AG

Email: AE@sensimed.ch

## Abnormal test findings

Abnormal baseline values and conditions will not be considered as adverse events. Any change in test findings and pre-existing conditions during the investigation will be considered adverse events and reported as such, provided that they are judged clinically significant by the investigator and unless an expected evolution of the condition in question.

## Causality and severity assessment

The investigator will promptly review documented adverse events (AE) and abnormal test findings to determine 1) if the abnormal test finding should be classified as an adverse event; 2) if there is a reasonable possibility that the adverse event was caused by the investigational device; and 3) if the adverse event meets the criteria for a serious adverse event.

For each adverse event, the investigator will evaluate the causality relationship between the investigational device and the observed adverse event. The relationship between the adverse event and the investigational device will be recorded as either of the following:

- Unrelated – the AE has no temporal (timing) relationship to the intervention and is not related to the investigational device
- Unlikely related - the AE has no temporal relationship to the intervention and is unlikely related to the investigational device
- Possibly related - the AE has a reasonable temporal relationship to the intervention and may be related to the investigational device.
- Probably related - the AE has a reasonable temporal relationship to the intervention and is likely related to the investigational device.
- Definitely related - the AE has a reasonable temporal relationship to the intervention and is clearly related to the investigational device.

If the investigator’s determination of causality is “possibly related”, “probably related” or “definitely related”, the adverse event will be classified as associated with the use of the investigational device for reporting purposes. If the investigator’s determination of causality is “unrelated” or “unlikely related”, the event will be considered as not associated with the use of the investigational device.

Severity is rated according as mild, moderate or severe:

- Mild: Symptom(s) barely noticeable to the patient or does not make the patient uncomfortable. The AE does not influence performance or functioning. Prescription drugs are not ordinarily needed for relief of symptom(s).
- Moderate: Symptom(s) of a sufficient severity to make the patient uncomfortable. Performance of daily activities is influenced. Treatment of symptom(s) may be needed.
- Severe: Symptom(s) of a sufficient severity to cause the patient severe discomfort. Severity may cause cessation of treatment with the drug. Treatment for symptom(s) may be given.

## Reporting Period

Complaints and adverse events are collected on an ongoing basis from baseline visit.

All new complaints and AEs must be recorded until the last study visit.

New protocol related complaints and AEs (caused by any intervention required by the protocol) and updates on AEs with an ongoing or unknown outcome must be recorded until the last subject visit required by the protocol. Beyond this reporting period, any new unsolicited serious AE spontaneously reported to the sponsor by the investigator would however be collected and processed.

Within a study, all patients who were exposed to the SENSIMED Triggerfish^®^ - whether they completed the recording session or not - should enter the complaint and AE recording period as defined above.

If a subject is documented as lost-to follow-up, ongoing/unknown outcome AEs will not be followed-up.

All ongoing/unknown outcome AEs will be followed-up until the last study visit. A last batch of queries will be sent after last study visit if remaining ongoing/unknown outcomes of reported AEs are pending.

After the last batch of queries with all collected data have been fully processed, CRFs and the database will no longer be updated. Only SAEs and medically relevant ongoing/unknown outcome AEs will be followed-up until resolution or stabilization or until 30 days after the subject was last exposed to the investigational device, whichever comes first.

# Statistical section

## Study Objectives

The objectives of this study are to assess the safety and effectiveness of the SENSIMED Triggerfish^®^ (TF) device in continuous recording of relative fluctuation in intraocular pressure (IOP). Safety will be assessed by recording of AEs during the 24 hours of continuous TF monitoring. TF efficacy will be evaluated by demonstrating TF ability to detect:

1. The known phenomenon of increase in IOP when moving from waking state to going to bed, as reflected in pneumotonometer measurements
2. Ocular Pulse frequency relative to direct measurement of Heart Rate (HR).

## Study Design

This is a single-center, single arm, open label study with subjects serving as their own control.

- Subjects will be monitored in a sleep laboratory with TF over 24 hours. All subjects will arrive at a similar time of day.
- Three (3) times during sleep HR will be measured directly over periods of six (6) minutes each. This duration has been chosen since TF activates every five (5) minutes for a single minute, and time of activation cannot be known until device recordings are examined. Thus the interval specified will ensure at least one, continuous thirty-second TF recording in parallel to HR measurement. Time of HR measurement will be logged and synchronized along with TF recording so that the two outputs can be compared at the same time points.
- Each subject will complete a diary of activities during the study. Of particular importance is the time at which subject went to bed at night.

## Population

Study population will consist of males and females whose inclusion and exclusion criteria are described in section 6.1.

## Overview of Investigation

Study procedures described in Section ‎1.2 are designed to evaluate TF's ability to:

- Detect changes in IOP from waking to sleep, based on the established phenomenon that IOP increases from waking to sleep hours.
- Accurately record the Ocular Pulse, which varies with arterial pulse.^[[2]](#footnote-2)^

### IOP Change from Wake to Sleep States

Relative fluctuations in IOP associated with going from waking state to sleep will be assessed by:

- Pneumotonometer every two hours from 6:00 pm until two IOP measurements have been taken after subject has gone to bed, with the first measurement at least ½ hour after having gone to bed
- Continuously with TF

Change in IOP from wake to sleep as measured by tonometer will be computed by:

1. Averaging IOP of the two tonometer measurements prior to going to sleep
2. Averaging IOP of the two tonometer measurements subsequent to going to sleep

Only subjects with average waking IOP at least 3 mmHg lower than sleep IOP will be included in the analysis. Difference between wake and sleep will be measured by subtracting average of the last two tonometer measures before going to bed from the average of the first two tonometer measurements subsequent to going to bed. Our aim then is to demonstrate a significant increase in TF-measured IOP in those subjects who displayed a meaningful difference on a standard tonometer.

It should be noted that 3 mmHg was chosen as the minimally important difference (MID), based on typical repeatability standard deviations of tonometers that vary between 2.5 mmHg and 4 mmHg^[[3]](#footnote-3)^. Assuming repeatability is about 3.0 mmHg, a change of 3 mmHg has about an 85% chance of reflecting true change in IOP.

### Ocular Pulse

As described in the Section ‎1.2, HR will be recorded three times during the night while TF is installed and recording continuously. Using these data we will relate Ocular Pulse as measured by TF to HR recorded directly.

## Endpoints

### Safety

Adverse events (AE's) including serious AE's (SAE's) occurring at any time during the trial or follow up, whether or not deemed related to study device. Safety measurements will include standard slit-lamp examination.

### Primary Efficacy

This study has the following two, co-primary TF endpoints for each subject:

1. Linear correlation between Time and Continuous TF recordings from approximately 4 hours preceding going to bed to 4 hours after. Relationship between TF and Time will be obtained by modeling the former against the latter using a second order polynomial^[[4]](#footnote-4)^. Linear correlation between TF and Time is the standardized β obtained from the model.
2. Percent difference between frequency of ocular pulsation and HR scored dichotomously:
   - Accurate = "Yes," if
   - Accurate = "No" otherwise

Ocular Pulse will be computed from TF either manually blinded to HR or analytically (e.g. Fast Fourier Transform). Fifteen percent (15%) difference between TF and HR measurements is specified because of the expected events, such as eye and lid movements, which introduce noise to TF measurement.

### Secondary Efficacy

Secondary efficacy endpoints include, for each subject:

- Subject Acceptance of TF, which will be measured on a 5 point analog scale where 5, indicates high acceptance of TF and 1 indicates low acceptance.
- Categorization of subject diurnal rhythm of IOP as measured by TF and assignment to pre-specified diurnal rhythms identified from the literature.

### Performance

- Validity of TF recording at the interval and subject levels scored dichotomously:
  - 0 – invalid
  - 1 – valid

## Analysis Sets

### Safety Analysis Set

The safety analysis set will consist of all patients for whom a recording with TF was initiated.

### Primary Efficacy Analysis Set

The primary efficacy analysis set will consist of all subjects with no major entry violations, as determined by blind review.

Subjects analyzed for Ocular Pulse must have valid data on TF and HR concurrently.

Subjects analyzed for wake/sleep change in IOP must have:

- A least one valid tonometer measure prior to going to bed and one subsequent, and at least 80% valid TF data before going to bed and 80% subsequent to going to bed.
- Difference between average tonometer measurement prior and subsequent to going to bed of 3 mmHg (or single tonometer measurement pre and/or post as available).

Treatment of Missing data:

For both TF and Ocular Pulse only observed data will be used. At the same time, modeling of TF wake/sleep data will be done over the whole wake/sleep period in the presence of up to 20% missing data. In essence this means that missing TF data will be imputed using model fitted to observed subject data.

### Performance Analysis Set

The performance analysis set will consist of all patients for whom a TF recording was initiated.

## Sample Size Considerations

This study has two co-primary endpoints so that presentation of sample size is based on demonstrating:

- A significant, positive and linear correlation between TF recordings and time during the wake/sleep period.
- Proportion of accurate TF Ocular Pulsation recording relative to HR (TF within ± 15% of HR) is at least 70%; i.e. 70% is an objective performance criterion (OPC) for demonstrating accuracy of Ocular Pulsation

We wish to have at least 90% power to show both of the above, implying 95% power for rejecting each null hypothesis (0.95 * 0.95 = 0.90, conservatively assuming independence of measurements done for testing the two hypotheses).

Previously collected data provide our best estimate for true linear relationship between TF and Time at 0.708 with a standard deviation of 0.285. After Fisher-z transformations of individual correlations we obtain a mean of 1.073 with standard deviation of 0.544. Given these estimates and using a one-sample t-test versus a null of no correlation, a sample size of 10 provides over 95% power to reject the null for this hypothesis.

At this stage we estimate that on 90% of one-minute thirty-second recordings Ocular Pulse as detected by TF will be within ± 15% of HR measured directly. To demonstrate 0.90 ≥ 0.70 with 95% power requires 53 independent observations using two-sided Exact Binomial confidence interval. In this study, each subject will contribute three (3), one-minute TF_Ocular Pulse_/HR relationships. While within-subject TF-HR agreement is not likely to be independent, we assume at this stage that 90 such observations, obtained from 30 subjects, will provide requisite power; i.e. will provide the equivalent of at least 53 independent observations. As described below, confidence interval will be computed using a mixed logistic model (SAS^®^ PROC GLIMMIX) to account for dependencies in the data. Simulations will be used to assess the assumption that 90 observations from 30 subjects provide at least 95% power. If simulations will indicate that more than 30 subjects are needed, this protocol will be amended accordingly before initiation of trial.

As noted, only subjects whose IOP measurements prior to going to bed and after differ by at least 3 mmHg will be analyzed for wake/sleep difference. Thus subjects will be evaluated on IOP change by tonometer and should some display a lower than 3 mmHg difference, additional subjects will be included until 30 valid subjects for testing IOP increase have been obtained. Evaluation of IOP change on tonometer will be done during the trial before (and blind to) opening of TF data. Subjects not included for testing change in IOP will however be included for testing the hypothesis relating to Ocular Pulse.

In conclusion, at this stage we estimate that 30 completed subjects will participate in this trial. This number may increase depending on simulation results and/or evaluation of subject validity for testing change in IOP from before to after going to bed.

## Statistical Analysis

### Overview

Safety, subject disposition and performance will be conducted on the safety analysis set. Primary and secondary efficacy will be conducted on the efficacy analysis set.

The data will be summarized in tables listing the mean, standard deviation median, minimum, maximum and number of subjects for continuous data, or in tables listing count and percentage for categorical data where appropriate. Tables will be presented by study arm and overall. Data listing by subject will be provided.

All statistical analyses will be performed and data appendixes will be created using the SAS^®^ system. The effects of noncompliance, dropouts, and possible covariates such as Age and Gender will be assessed to determine the impact on the general applicability of results from this study.

### Subject Disposition

Subject disposition will be tabulated; the number of enrolled, exposed, prematurely terminated and completed subjects will be summarized.

A list of dropouts will be prepared including reason for discontinuation, and time of discontinuation. In addition, disposition with regard to replacement will be provided.

### Safety

The safety analyses will be descriptive and narrative in nature, with AEs, including serious AEs (SAEs), coded using MedDRA and tabulated by body system, preferred term, group, severity and relation to procedure. Descriptive statistics and shift analysis tables will be provided as appropriate for Ophthalmological examination results.

### Primary Efficacy

#### Wake/Sleep Correlation

Stage 1: Assessing Validity of Subjects

As noted, only subjects with IOP increases of at least 3 mmHg from wake to sleep, as measured by hand-held tonometer will be included. Thus, Stage 1 of this analysis will consist of evaluating IOP differences between wake and sleep to determine those subjects who will be included in this primary efficacy analysis.

Stage 2: Testing of co-primary endpoint

The following hypotheses will be tested

H_0_: ρ = 0

H_1_: ρ ≠ 0

Where ρ is the correlation between TF and Time over 8 hours, the first four being in a waking state and the subsequent 4, after going to bed.

The relationship between TF and Time over the wake/sleep period will be modeled with the following equation:

TF_i_ = C + B_1_Time_i_ + B_2_Time_i_^2^

Where,

TF_i_ – TF recording at Time i

B_1_ – Weight of linear term (linear slope)

B_2_ – Weight of quadratic term

The statistical hypotheses will be tested by:

- Obtaining for each subject β for the linear term from the model (i.e. the standardized B of the linear term)
- Transforming each β using Fisher-z
- Comparing the mean z-transformed correlation to 0 using a one-sample t-test with two-sided alpha=0.05.

We will conclude success on this primary endpoint if the correlation is significantly greater than 0; i.e. positive and significant.

#### Accuracy of Ocular Pulse

In the first stage of this analysis each instance of parallel HR and TF recording will be coded as 1 (“success;” Ocular Pulse as measured by TF is within ± 15% of HR measured directly) or 0 (“failure;” otherwise). Assessment of success or failure will be done by comparing HR measured directly to the thirty-second TF recording in the interval HR was measured. If two TF recordings are obtained during the six (6) minutes of direct HR recording, that most overlapping with the HR recording will be chosen. If both TF measurements show the same overlap with HR, both will be used.

The second co-primary endpoint will then be tested via the following hypotheses:

H_0_: π_accuary_ < 0.70

H_1_: π_accuary_ ≥ 0.70

Where,

π_accuary_ – is the proportion of Ocular Pulse values for one minute intervals that are within ± 15% of HR measured directly

Hypotheses will be tested by constructing a 95% two-sided confidence interval using SAS^®^ PROC GLIMMIX (logistic link function) to account for possible dependency between measurements taken from the same individual.

### Secondary Efficacy

Diurnal Rhythm: Prior to the trial, 24-hour diurnal IOP rhythm types will be identified in the literature. Evaluators will then be trained to categorize subjects’ 24-hour TF recording into these rhythms types. A protocol for this training, and statistics used for its evaluation (e.g. Kappa for agreement between raters), will be presented separately.

Each subject in the trial with at least 80% valid TF data will be categorized as having one of the diurnal IOP rhythms by two independent raters. Rater agreement will be described using a K x K confusion matrix, K being the number of rhythm types identified. Cohen’s Kappa will be computed for this table and presented along with two-sided 95% confidence intervals.

Subject Acceptance: Subject Acceptance of TF will be measured by questionnaire and presented descriptively, including mean along with associated two-sided 95% confidence interval.

### Performance

Validity rate of TF measurements will be computed per subject. Descriptive statistics will then be provided over all subjects, including two-sided 95% confidence intervals for the mean.

## Interim Analysis

No interim analysis is planned in this trial.

# scientific analysis of the protocol

SENSIMED Triggerfish^®^ is a device intended for continuous IOP fluctuation monitoring. The system consists of several parts, of which the Sensor is based on a soft silicone contact lens integrating microelectronic components. A circular strain gage senses variations in central corneal curvature that have been shown to be related to changes in IOP. Like with tonometers that detect IOP through surrogate variables, there is a need to determine how the surrogate variable relates to the monitored property.

Leonardi et al have shown excellent linearity and repeatability during monitoring of fluctuating IOP in pressure-controlled, enucleated porcine eyes. In enucleated eye, however, whether animal or human, corneal properties such as elasticity and hydration status are significantly altered compared to in vivo eyes. Phenomena such as eye blinks and circadian CCT variation are absent. Further, appropriate corneal hydration and post-lens liquid film cannot be maintained artificially for periods extending beyond a few minutes. Specific investigation of the relationship between IOP and SENSIMED Triggerfish^®^ output signal is therefore of interest in vivo.

It is the purpose of this investigation to correlate IOP values obtained with a conventional tonometer to the output provided by SENSIMED Triggerfish^®^.

While IOP cannot be measured on an eye with the SENSIMED Triggerfish^®^, it *can* be measured on the fellow eye. For this to be meaningful there needs to be a relationship between the two eyes in glaucoma patients. Studies show that while the correlation in IOP between fellow eyes is far from perfect, it is clinically meaningful. Specifically, Dinn et al report that about 76% of fellow eyes were, on average in a given 3-hour interval, within 2mmHg of each other; 87% were within 3mmHg of each other. Dinn et al conclude that "diurinal IOP profiles between fellow eyes are parallel in POAG patients." Moreover, the similarity is greater in treated relative to untreated patients. They do however concede that this relationship has its limitations and that in up to 20% of treated patients there will be an asymmetric relationship between fellow eyes of 3mmHg or more. It is however important to note that 3mmHg is within the error or measurement of GAT.

Sit et al report a weaker relationship to that described in the preceding paragraph. At the same time, they found that correlation between fellow eyes under different conditions (e.g. office-hour sitting, diurnal supine) ranged between 0.65 and 0.73 (mean r = 0.70) and was significant. Additionally, about 85% of fellow eyes were within 3mmHg on a given measurement time point. Sit et al divided the day into 4 periods and computed mean, peak and trough IOP values for each. When averaged over all subjects, fellow eyes never differed by more than 1mmHg for any of these parameters.

In conclusion, it appears that fellow eyes can provide reasonable surrogate IOP measurements for one another.

Based on the above considerations, we consider this investigation as scientifically sound.

# Administrative and legal aspects

## Approval of Trial Protocol and Amendments

Before the start of the trial, the trial protocol, informed consent document, and any other appropriate documents will be submitted to the independent registered EC or IRB. Formal approval by the EC or IRB should preferably mention the title of the trial, the trial code, the trial site, and any other documents reviewed. It must mention the date on which the decision was made and must be officially signed by a committee member.

Before the first subject is enrolled in the trial, all ethical and legal requirements must be met.

Neither the investigator nor the sponsor will alter this trial protocol without obtaining the written agreement of the other. The EC or IRB must be informed of all protocol amendments.

The investigator must keep a record of all communications with the EC or IRB.

## Continuous Information to competent Ethics Committee

The EC or IRB must be informed of all subsequent protocol amendments which require formal approval in accordance with local legal requirements.

The EC or IRB must be informed of serious adverse device effects, under the responsibility of the investigator, if not otherwise stated in the vote.

The EC or IRB must be informed regularly of the trial process, if not otherwise stated in the vote. The EC or IRB must be informed at the end of the trial.

Any additional requirements imposed by the EC or IRB will be followed.

## Submission to Regulatory Authorities

This investigation will be submitted to regulatory authorities for review and approval if required by local applicable requirements. Safety reporting will be done according to applicable legal requirements.

## Quality assurance

The procedures set out in this trial protocol, pertaining to the conduct, evaluation, and documentation of this trial, are designed to ensure that all persons involved in the trial abide by ISO 14155:2003 and the ethical principles described in the current revision of the Declaration of Helsinki. The trial will be carried out in keeping with local legal (California) and regulatory requirements.

## Informed consent

Prior to any other investigation related procedures, informed consent will be obtained from patients after they have received oral and written information concerning the investigation. Informed subjects will be provided time to consider their participation in the investigation and will be given the possibility to inquire about details of the investigation. Consent forms will be signed and dated personally by the patient and the investigator. Patients will receive copies of the written information and of the signed consent forms.

Should new information become available during the investigation that may affect the patients’ decision to participate, this new information will be made available to the patients in written and oral form by the investigator. When appropriate, patients will be asked to sign an updated consent form if they wish to continue participating.

## Insurance

Insurance coverage for the patients in the current study is provided by Sensimed AG.

## Responsibilities of Investigator

The investigator and sponsor should ensure that all persons assisting with the trial are adequately informed about the protocol, any amendments to the protocol, the trial treatments, and their trial-related duties and functions.

The investigator is not allowed to deviate from the protocol, unless the deviation has as purpose to protect subjects rights, safety and well-being. All protocol deviations will be recorded on the appropriate form.

This is a company-sponsored investigation. The investigator will be responsible for maintaining and updating the investigator site file.

The investigator and the Sponsor should maintain a list of subinvestigators and other appropriately qualified persons to whom the investigator has delegated significant trial-related duties. Logs containing information on all patients who signed an informed consent, their investigation ID (anonymized) and their corresponding contact data will be maintained throughout the investigation as well. The attribution of SENSIMED Triggerfish^®^ (Sensor, Antennae, Recorder and Data Cable) will be recorded for all enrolled patients on the appropriate forms.

The final report will be prepared by Sensimed.

## Data Management

Investigational data will be recorded on CRFs. CRFs will be filled for enrolled patients only (not screening failures). Through his/her signature on the CRF, the investigator will certify that the data collected for each patient are accurate, complete and legible.

All data generated in the current clinical investigation will be managed according to standard procedures. Data and other study documentation will be archived for a minimum of 10 years after the completion of the investigation. Written approval from the sponsor should be obtained prior to the destruction of any investigation related documents.

## Confidentiality

During the clinical trial, subjects will be identified solely by means of their individual identification code (subject number, randomized number). Trial findings stored on a computer will be stored in accordance with local data protection law and will be handled in the strictest confidence. For protection of these data, organizational procedures are implemented to prevent distribution of data to unauthorized persons. The appropriate regulations of local data legislation will be fulfilled in its entirety.

All patient data will be recorded on a patient-specific Case Report Form (CRF). Patient identification will be done by a unique patient code. Authorized persons may inspect the subject-related data collected during the trial ensuring the data protection law as well as the accuracy and legibility of the recorded data.

The investigator will maintain a personal subject identification list (subject numbers with the corresponding subject names) to enable records to be identified.

## Early termination or Suspension of the Investigation

The sponsor may terminate the investigation prematurely in the case of non-compliance of the investigational site(s) or in case of safety concerns.

## End of the Investigation and Final Report

The end of the investigation (EOI) is defined as the last patient off study. The final report is written by the sponsor.

## Publication Policy

The results of this clinical investigation will be offered for publication by the investigator(s). The Coordinating Clinical Investigator will be the corresponding first author of such a publication. In the case of several study centers, centers’ Principal Clinical Investigators will appear in the author list in ranked manner, as a function of their patient recruitment in decreasing order, and the Principal Coordinating Investigator will appear on the publication as the corresponding first author.

Publication of the study results is subject to written approval by Sensimed, following a review period of all materials to be published of up to 20 days. Publication of the final results will not be denied by without reasonable grounds by Sensimed.

1. risk analysis

# risk analysis

## Anticipated clinical benefits

There may be no direct benefit to the patients participating in this investigation. Patients will receive an ophthalmic examination free of charge.

Glaucoma is the second cause of blindness worldwide. The only risk factor for glaucoma that can be manipulated in the clinical setting is IOP. No available technology can perform uninterrupted 24-hour IOP measurements during sleep or during daily activities. Consequently, the ophthalmologist does not have the tool that permits widespread, cost-effective, and precise recording of the IOP for the full 24-hour period, in the case of Glaucoma patients or for Glaucoma screening. As a result, the disease remains under-diagnosed and many Glaucoma patients are kept on insufficient or inappropriate medication.

The validation of a tool like SENSIMED Triggerfish^®^ may benefit to the larger glaucoma and glaucoma suspect population and lead to better disease and patient management.

## Anticipated risks

### SENSIMED Triggerfish^®^

The risks correspond to the risks caused by wearing commercially available soft contact lenses. These risks include pain, redness of the eye, discomfort, conjunctival and corneal infections, dry eye sensations and swelling of the cornea.

An ophthalmological examination of the eye is done prior to Sensor placing to exclude risk factors that could place the patient at increased risk for ocular complications and after Sensor removal for appropriate detection and treatment of any device-related side effects. Patients will be provided a direct medical contact that can be reached throughout each IOP fluctuation recording session with SENSIMED Triggerfish^®^.

The tape with antenna may cause local skin irritation or, if used on very sensitive skin, local inflammation. Known or unknown allergy to any material in the antenna can provoke an allergic response. During removal of the antenna, some hair may be epilated. Very rarely electrical dysfunction in the antenna or a damaged connector can induce a mild electrical shock.

In order to minimize these risks, the device is manipulated only by qualitifed health care professionals who have been trained in the manipulation and operation of SENSIMED Triggerfish. The antenna is applied on clean skin and patients are instructed not to manipulate the SENSIMED Triggerfish^®^ system during the recording session.

Anticipated risks are detailed in Part L. The SENSIMED Triggerfish^®^ Directions For Use also contains a list of anticipated complications.

### Tonometer

Repeated IOP measurements using any contact tonometer, such as GAT or pneumatonometer, may put the patient at increased risk for ocular complications mainly due to the contact between the tonometer and the cornea. The ocular anesthetic may also provoke side effects.

Anticipated risks are detailed in Part L.

## Benefit risk ratio

Patients will receive an ophthalmologic examination and an IOP profile over 24 hours. While there may be no direct benefit to patients in this investigation, a tool like the SENSIMED Triggerfish^®^ allows establishing a continuous profile of the IOP behaviour over the recorded period, giving the ophthalmologist the possibility to adapt a personalized medicine approach. Validating the safety and efficacy of the SENSIMED Triggerfish^®^ may also benefit to the larger glaucoma population, of which a certain portion experiences disease progression, despite well-controlled IOP as assessed during regular office visits or during IOP curves with traditional, punctual tonometry.

These points taken into consideration, the benefits associated with the conduct of the current investigation outweigh the risks and therefore justify conducting the investigation.

## Description of patient population

This investigation will enroll glaucoma patients and healthy subjects of either gender. Participants will be 18 to 80 years old but are expected to be mainly elderly.

1. Device description

# device description

## General

The SENSIMED Triggerfish^®^ is a break-through solution to record intraocular pressure fluctuation continuously for up to 24 hours to aid the diagnosis and treatment of glaucoma.

SENSIMED Triggerfish^®^:

- Is non-invasive
- Records IOP fluctuation continuously for up to 24 hours
- Functions during sleep and normal activities.

The patient wears the SENSIMED Triggerfish^®^ for 24 hours and assumes normal activities, including during sleep. At the end of the 24 hours, the data from the Recorder is transferred to the computer, where a qualified expert reviews and, if necessary, edits the data.

In case of purchasing a complete System, items 1-8 must be included:

The SENSIMED Triggerfish^®^ Set consists of:

1. SENSIMED Triggerfish^®^ Ocular Telemetry Sensor
2. SENSIMED Triggerfish^®^ Antenna
3. SENSIMED Triggerfish^®^ Recorder
4. SENSIMED Triggerfish^®^ Data Cable
5. SENSIMED Triggerfish^®^ Software
6. Recorder Sleeve
7. Battery Charger
8. Bluetooth USB (Universal Serial Bus) Stick

### SENSIMED Triggerfish^®^ Ocular Telemetry Sensor

The “Sensor” is a disposable silicone soft contact lens with a sensor embedded in it, allowing the measurement of changes in corneal curvature induced by intraocular pressure variation, wirelessly.

The Sensor has the following properties:

- Diameter: 14.1 mm
- Base Curves: 8.4 (STEEP), 8.7 (MEDIUM) and 9.0 mm (FLAT)
- Lens material: Silicone (polydimethylsiloxane (PDMS))
- Specific Gravity: 1.05
- Refractive Index: 1.43
- Light Transmission: Measures greater than 85% - dry
- Surface Character: Hydrophilic
- Water Content: Approximately 0.2%

### SENSIMED Triggerfish^®^ Antenna

The “Antenna” is the telemetry communication antenna for intraocular pressure fluctuation recording, comfortably cushioned with a soft breathable medical grade material and an elastic breathable tape. The Antenna is easy to apply and remove from the patient. The antenna is nickel-free.

### SENSIMED Triggerfish^®^ Recorder

The “Recorder” is a portable external unit, which contains the battery and is recording data. The integrated battery is rechargeable via a standard medical grade charger.

### SENSIMED Triggerfish^®^ Data Cable

The “Data Cable” connects the Antenna to the Recorder and is designed for data transmission during intraocular pressure fluctuation recording.

### SENSIMED Triggerfish^®^ Software

The “Software” is the support program to visualize the recorded data on the health care professional’s computer. It allows the health care professional to retrieve, view, and manage the recorded data in a standard format.

**Battery charger**

The medical grade battery charger is provided with the Recorder to charge the power into the battery of the Recorder.

**Sleeve**

Tyvek disposable sleeve is intended to be used with the recorder. It will protect the Recorder from damage and will allow patient free movements during day activities and during sleep.

## Therapeutic/diagnostic Effects

SENSIMED Triggerfish^®^ has no therapeutic or diagnostic effects.

## Device operation

The device is operated only by qualified health care professionals who have been trained by Sensimed representatives in the handling and operation of SENSIMED Triggerfish^®^.

All information concerning the device operation can be found in the SENSIMED Triggerfish^®^ User Manual, provided as a separate document.

## Manufacturer

SENSIMED Triggerfish^®^ is manufactured by:

Sensimed AG

Rue Centrale 6

1003 Lausanne

Switzerland

## Catalogue information

SENSIMED Triggerfish^®^ items are identified as shown below. SENSIMED Triggerfish^®^ is not authorized for sales in the USA.

| ST1001 | **SENSOR STEEP** | Ocular Telemetry Sensor – STEEP |
| --- | --- | --- |
| ST1002 | **SENSOR MEDIUM** | Ocular Telemetry Sensor – MEDIUM |
| ST1003 | **SENSOR FLAT** | Ocular Telemetry Sensor – FLAT |
| ST2001 | **ANTENNA – LEFT** | Telemetry communication antenna LEFT |
| ST2002 | **ANTENNA - RIGHT** | Telemetry communication antenna RIGHT |
| ST5000 | **RECORDER** | Portable data Recorder |
| ST5010 | **DATA CABLE** | Data cable connecting Recorder & Antenna |
| ST6000 | **CHARGER** | Battery charger |
| ST8000 | **SOFTWARE** | Software for data |
| ST9000 | **BLUETOOTH** | Bluetooth communication with PC |
| ST4001 | **SLEEVE** | Pocket for Recorder |

## Device traceability

Sensors, Recorders and Data Cables are assigned individual ID numbers in addition to lot numbers. Antennae and Sleeves carry a lot number and Software carries a revision number.

Complete device traceability will be ensured for all device components. The investigational device will be reconciled at the end of the investigation.

1. investigation monitoring

# monitoring procedures

This clinical investigation will be monitored to ensure that the rights and well-being of human subjects are protected, that the reported trial data are accurate, complete and verifiable from source documents and that the investigation is in compliance with the currently approved protocol/amendment(s), ISO 14155:2003 and applicable regulatory requirements. For monitoring purposes, direct access to trial-related records will be provided by the investigator.

Monitoring will be carried out by Sensimed AG at approximate monthly intervals. Contact details for the study monitor are provided below.

Lori Dismant, RN, CNOR, CCRA

**Manager, Clinical Monitoriing Services**

Located at:

Premier Research Group

1420 W. Kristal Way

Phoenix, AZ 85027

USA

Tel: +1 623 322 4301

Fax: +1 623 322 4300

Email: lori.dismant@premier-research.com

Detailed monitoring procedures are provided as a separate document.

1. labeling

In this investigation CE marked device is used, however, SENSIMED Triggerfish^®^ is not authorized for sales in the USA. All CE marked items carry approved labels as registered in Europe. Additionally, each item will be provided with a sticker indicating that the item is for investigation use only.

Battery chargers and Bluetooth adaptors are not Sensimed products. They are labeled with the CE mark by their manufacturer. Where not authorized for sales, any requirement concerning investigational device labeling will be complied with.

1. consent materials

Before being admitted to the clinical trial, the subject must consent to participate after the nature, scope, and possible consequences of the clinical trial have been explained in a form understandable to him or her. The subject must give consent in writing.

A copy of the signed informed consent document must be given to the subject. The documents must be in a language understandable to the subject and must specify who informed the subject.

The Subject Information and Subject Consent Form are provided as separate documents.

1. irb/EC information

The current investigation will be reviewed by:

University of California, San Diego

Human Research Protections Program

La Jolla Village Professional Center

8950 Villa La Jolla

La Jolla, California 92037

**USA**

President (chair person): Michael Caligiuri, PhD

1. other institutions

TechnoSTAT is involved in the current investigation providing statistical design and data analysis services.

TechnoSTAT Ltd.

34^th^ Jerusalem Street

Beit Gamla, 5^th^ floor

43501 Raanana

Israel

The investigation will be monitored by XX.

Premier Research Group

1420 W. Kristal Way

Phoenix, AZ 85027

USA

1. additional records and reports

None.

1. Literature

Asrani S, Zeimer R, Wilensky J, Gieser D, Vitale S, Lindenmuth K. [Large diurnal fluctuations in intraocular pressure are an independent risk factor in patients with glaucoma.](http://www.ncbi.nlm.nih.gov/pubmed/10782622?ordinalpos=2&itool=EntrezSystem2.PEntrez.Pubmed.Pubmed_ResultsPanel.Pubmed_DefaultReportPanel.Pubmed_RVDocSum) J Glaucoma. 2000 Apr;9(2):134-42.

[Bertsch A](http://www.ncbi.nlm.nih.gov/sites/entrez?Db=pubmed&Cmd=Search&Term=%22Bertsch%20A%22%5BAuthor%5D&itool=EntrezSystem2.PEntrez.Pubmed.Pubmed_ResultsPanel.Pubmed_DiscoveryPanel.Pubmed_RVAbstractPlus), [Leonardi M](http://www.ncbi.nlm.nih.gov/sites/entrez?Db=pubmed&Cmd=Search&Term=%22Leonardi%20M%22%5BAuthor%5D&itool=EntrezSystem2.PEntrez.Pubmed.Pubmed_ResultsPanel.Pubmed_DiscoveryPanel.Pubmed_RVAbstractPlus), [Renaud P](http://www.ncbi.nlm.nih.gov/sites/entrez?Db=pubmed&Cmd=Search&Term=%22Renaud%20P%22%5BAuthor%5D&itool=EntrezSystem2.PEntrez.Pubmed.Pubmed_ResultsPanel.Pubmed_DiscoveryPanel.Pubmed_RVAbstractPlus) The sensing contact lens. [Med Device Technol.](javascript:AL_get(this,%20'jour',%20'Med%20Device%20Technol.');) 2006 Jun

Dinn R, Zimmerman M, Shuba L, Doan A, Maley M, Greenlee E, Alward W and Kwon Y. Concordance of diurnal intraocular pressure between fellow eyes in primary open-angle glaucoma. Ophthalmology 2007 May;114(5):915-920.

[Fukano Y](http://www.ncbi.nlm.nih.gov/sites/entrez?Db=pubmed&Cmd=Search&Term=%22Fukano%20Y%22%5BAuthor%5D&itool=EntrezSystem2.PEntrez.Pubmed.Pubmed_ResultsPanel.Pubmed_DiscoveryPanel.Pubmed_RVAbstractPlus), [Kawazu K](http://www.ncbi.nlm.nih.gov/sites/entrez?Db=pubmed&Cmd=Search&Term=%22Kawazu%20K%22%5BAuthor%5D&itool=EntrezSystem2.PEntrez.Pubmed.Pubmed_ResultsPanel.Pubmed_DiscoveryPanel.Pubmed_RVAbstractPlus). Disposition and Metabolism of a Novel Prostanoid Antiglaucoma Medication, Tafluprost, Following Ocular Administration to Rats. [Drug Metab Dispos.](javascript:AL_get(this,%20'jour',%20'Drug%20Metab%20Dispos.');) 2009 May 28.

[Hughes E](http://www.ncbi.nlm.nih.gov/sites/entrez?Db=pubmed&Cmd=Search&Term=%22Hughes%20E%22%5BAuthor%5D&itool=EntrezSystem2.PEntrez.Pubmed.Pubmed_ResultsPanel.Pubmed_DiscoveryPanel.Pubmed_RVAbstractPlus), [Spry P](http://www.ncbi.nlm.nih.gov/sites/entrez?Db=pubmed&Cmd=Search&Term=%22Spry%20P%22%5BAuthor%5D&itool=EntrezSystem2.PEntrez.Pubmed.Pubmed_ResultsPanel.Pubmed_DiscoveryPanel.Pubmed_RVAbstractPlus), [Diamond J](http://www.ncbi.nlm.nih.gov/sites/entrez?Db=pubmed&Cmd=Search&Term=%22Diamond%20J%22%5BAuthor%5D&itool=EntrezSystem2.PEntrez.Pubmed.Pubmed_ResultsPanel.Pubmed_DiscoveryPanel.Pubmed_RVAbstractPlus). 24-hour monitoring of intraocular pressure in glaucoma management: a retrospective review [J Glaucoma.](javascript:AL_get(this,%20'jour',%20'J%20Glaucoma.');) 2003 Jun

Kim M, Kim J, Park K and Choi C. Assymmetry of diurnal intraocular pressure fluctuation between right and left eyes. Acta Ophthalmol. 2009 Oct (e-pub ahead of print).

[Leonardi M](http://www.ncbi.nlm.nih.gov/sites/entrez?Db=pubmed&Cmd=Search&Term=%22Leonardi%20M%22%5BAuthor%5D&itool=EntrezSystem2.PEntrez.Pubmed.Pubmed_ResultsPanel.Pubmed_DiscoveryPanel.Pubmed_RVAbstractPlus), [Leuenberger P](http://www.ncbi.nlm.nih.gov/sites/entrez?Db=pubmed&Cmd=Search&Term=%22Leuenberger%20P%22%5BAuthor%5D&itool=EntrezSystem2.PEntrez.Pubmed.Pubmed_ResultsPanel.Pubmed_DiscoveryPanel.Pubmed_RVAbstractPlus), [Bertrand D](http://www.ncbi.nlm.nih.gov/sites/entrez?Db=pubmed&Cmd=Search&Term=%22Bertrand%20D%22%5BAuthor%5D&itool=EntrezSystem2.PEntrez.Pubmed.Pubmed_ResultsPanel.Pubmed_DiscoveryPanel.Pubmed_RVAbstractPlus), [Bertsch A](http://www.ncbi.nlm.nih.gov/sites/entrez?Db=pubmed&Cmd=Search&Term=%22Bertsch%20A%22%5BAuthor%5D&itool=EntrezSystem2.PEntrez.Pubmed.Pubmed_ResultsPanel.Pubmed_DiscoveryPanel.Pubmed_RVAbstractPlus), [Renaud P](http://www.ncbi.nlm.nih.gov/sites/entrez?Db=pubmed&Cmd=Search&Term=%22Renaud%20P%22%5BAuthor%5D&itool=EntrezSystem2.PEntrez.Pubmed.Pubmed_ResultsPanel.Pubmed_DiscoveryPanel.Pubmed_RVAbstractPlus) First steps toward noninvasive intraocular pressure monitoring with a sensing contact lens [Invest Ophthalmol Vis Sci.](javascript:AL_get(this,%20'jour',%20'Invest%20Ophthalmol%20Vis%20Sci.');) 2004 Sep

[Leonardi M](http://www.ncbi.nlm.nih.gov/sites/entrez?Db=pubmed&Cmd=Search&Term=%22Leonardi%20M%22%5BAuthor%5D&itool=EntrezSystem2.PEntrez.Pubmed.Pubmed_ResultsPanel.Pubmed_DiscoveryPanel.Pubmed_RVAbstractPlus), [Pitchon EM](http://www.ncbi.nlm.nih.gov/sites/entrez?Db=pubmed&Cmd=Search&Term=%22Pitchon%20EM%22%5BAuthor%5D&itool=EntrezSystem2.PEntrez.Pubmed.Pubmed_ResultsPanel.Pubmed_DiscoveryPanel.Pubmed_RVAbstractPlus), [Bertsch A](http://www.ncbi.nlm.nih.gov/sites/entrez?Db=pubmed&Cmd=Search&Term=%22Bertsch%20A%22%5BAuthor%5D&itool=EntrezSystem2.PEntrez.Pubmed.Pubmed_ResultsPanel.Pubmed_DiscoveryPanel.Pubmed_RVAbstractPlus), [Renaud P](http://www.ncbi.nlm.nih.gov/sites/entrez?Db=pubmed&Cmd=Search&Term=%22Renaud%20P%22%5BAuthor%5D&itool=EntrezSystem2.PEntrez.Pubmed.Pubmed_ResultsPanel.Pubmed_DiscoveryPanel.Pubmed_RVAbstractPlus), [Mermoud A](http://www.ncbi.nlm.nih.gov/sites/entrez?Db=pubmed&Cmd=Search&Term=%22Mermoud%20A%22%5BAuthor%5D&itool=EntrezSystem2.PEntrez.Pubmed.Pubmed_ResultsPanel.Pubmed_DiscoveryPanel.Pubmed_RVAbstractPlus). Wireless contact lens sensor for intraocular pressure monitoring: assessment on enucleated pig eyes. [Acta Ophthalmol.](javascript:AL_get(this,%20'jour',%20'Acta%20Ophthalmol.');) 2009 Jun;87(4):433-7

McNamara N, Polse K, Brand R, Graham A, Chan J and McKenney C. Tear mixing ounder a sift contact lens: effects of lens diameter. Am J Ophthalmol 1999 Jun(127)6:659-665.

Miller K, Polse K and Radke C. Fenestrations enhance tear mixing under silicone-hydrogel contact lenses. Invest Ophthalmol Vis Sci 2003 Jan

[Pierscionek BK](http://www.ncbi.nlm.nih.gov/sites/entrez?Db=pubmed&Cmd=Search&Term=%22Pierscionek%20BK%22%5BAuthor%5D&itool=EntrezSystem2.PEntrez.Pubmed.Pubmed_ResultsPanel.Pubmed_DiscoveryPanel.Pubmed_RVAbstractPlus), [Asejczyk-Widlicka M](http://www.ncbi.nlm.nih.gov/sites/entrez?Db=pubmed&Cmd=Search&Term=%22Asejczyk-Widlicka%20M%22%5BAuthor%5D&itool=EntrezSystem2.PEntrez.Pubmed.Pubmed_ResultsPanel.Pubmed_DiscoveryPanel.Pubmed_RVAbstractPlus), [Schachar RA](http://www.ncbi.nlm.nih.gov/sites/entrez?Db=pubmed&Cmd=Search&Term=%22Schachar%20RA%22%5BAuthor%5D&itool=EntrezSystem2.PEntrez.Pubmed.Pubmed_ResultsPanel.Pubmed_DiscoveryPanel.Pubmed_RVAbstractPlus) The effect of changing intraocular pressure on the corneal and scleral curvatures in the fresh porcine eye [Br J Ophthalmol.](javascript:AL_get(this,%20'jour',%20'Br%20J%20Ophthalmol.');) 2007 Jun

[Sit AJ](http://www.ncbi.nlm.nih.gov/sites/entrez?Db=pubmed&Cmd=Search&Term=%22Sit%20AJ%22%5BAuthor%5D&itool=EntrezSystem2.PEntrez.Pubmed.Pubmed_ResultsPanel.Pubmed_DiscoveryPanel.Pubmed_RVAbstractPlus), [Liu JH](http://www.ncbi.nlm.nih.gov/sites/entrez?Db=pubmed&Cmd=Search&Term=%22Liu%20JH%22%5BAuthor%5D&itool=EntrezSystem2.PEntrez.Pubmed.Pubmed_ResultsPanel.Pubmed_DiscoveryPanel.Pubmed_RVAbstractPlus) Pathophysiology of glaucoma and continuous measurements of intraocular pressure. : [Mol Cell Biomech.](javascript:AL_get(this,%20'jour',%20'Mol%20Cell%20Biomech.');) 2009 Mar

Sit A, Liu J and Weinreb R. Asymmetry of right versus left intraocular pressures over 24 hours in glaucoma patients. Ophthamology, 2006 March. 113(3):425-430.

1. anticipated adverse events

# adverse device effects and complications

## Related to SENSIMED Triggerfish^®^ Sensor

Ocular Telemetric Sensor for continuous, non invasive IOP fluctuation recording for up to 24 hours.

**Discomfort**

Any feeling of discomfort to the patient either from wearing, inserting, or removing the lens. Can manifest itself as: itching, irritation, swelling, impaired vision, difficulty to perform daily tasks, difficulty to sleep.

**Contact Lens Related Allergy**

Contact allergic conjunctivitis occurs when the contact lenses themselves or the proteins in tear film that bind to the surface of the lens can cause an irritative response of the conjunctiva, resulting in redness, itching, mucous discharge, and lens discomfort.

**Dry eyes**

Dry eyes are common in those who wear contact lenses. They affect some soft-lens wearers and many hard-lens wearers. Eye diseases and other diseases and certain medicines can cause dry eyes. Symptoms of dry eyes include: foreign body sensation, tearing or burning (or both), dryness in the eye (a hot, sandy feeling), redness.

Edema

Abnormal accumulation of fluid beneath the skin in association with allergy, inflammation or infection.

**Epiphora**

Excessive tear production. A result of irritation, allergy or infection.

**Eye injury**

Physical or chemical injuries of the eye can be a serious threat to vision if not treated appropriately and in a timely fashion. Eye injury can come from a blow, scratch, or foreign object).

Foreign body sensation

The sensation that something is in the eye, as if a foreign body were scratching the eye. Usually this sensation originates from the cornea with its large number of nerve fibers.

Hyperaemia

Describes the increase of blood flow to body tissue.

**Allergy to silicon or Allergic reactions to contact lens solution**

Hypersensitivity to a material can demonstrate as itching, burning, reddening or swelling.

**Itching, burning, or gritty feeling**

Belong to irritation, inflammation, infection, allergy, injury.

**Visual acuity reduction**

Lack of sharpness of vision with, as a result, the inability to see fine detail. Blurred vision can occur when a person who wears corrective lens is without them. Blurred vision has 4 general mechanisms: opacity of normally transparent ocular structures, retinal disorders, disorders affecting the optic nerve or its connections and refractive errors.

**Swelling**

Belongs to allergy, inflammation, infection.

**Pain**

Pain in the eye (that is not due to injury) may be described as a burning, throbbing, aching, or stabbing sensation in or around the eye. It may also feel as if there is a foreign body in the eye.

**Conjunctivitis**

Conjunctivitis is an inflammation or an infection of the conjunctiva or thin membrane covering the white of the eye and the inner surface of the eyelid. The inflamed/infected conjunctiva will usually make the eye appear red or pink because the tiny blood vessels that are normally within the conjunctiva are now irritated and enlarged.

**Corneal abrasion**

A corneal erosion or abrasion can occur when the cornea is scraped or injured. In these cases, there is localised loss of the corneal epithelium, the cornea's outer layer. These painful conditions quite commonly arise after a poke from a baby's fingernail or tree limbs and bushes, or vigorous rubbing of the eye. Sometimes they are caused by contact lenses.

**Corneal staining**

Appears as fluorescein-positive spots when observed at the slit lamp. Rather than corneal lesions, the observed spots indicate a temporary difference in epithelial structure without affecting epithelial integrity.

**Eye irritation**

An excessive response to stimulation in an eye or eyelid, a condition of soreness or inflammation

**Corneal ulcers**

A corneal ulcer, or ulcerative keratitis, is an inflammatory or more seriously, infective condition of the cornea involving disruption of its epithelial layer with involvement of the corneal stroma.

**Eye infection**

Infection of the cornea is called infectious keratitis. It is the most serious complication of contact lens wear and can cause blindness. Bacterial keratitis is the most common type of infectious keratitis. Overall, bacterial keratitis is among the least frequent complications of wearing contacts, but it is much more common in people with extended-wear soft lenses, especially those who wear the lenses overnight.

On rare occasions, viruses and fungi may cause keratitis. A germ that is often present in tap water causes a form of keratitis that is increasingly seen in contact lens wearers, especially those with soft lenses.

## Related to SENSIMED Triggerfish^®^ Antenna

**Discomfort**

Patient can experience a discomfort of wearing the patch on the face skin. Discomfort defined as itching, pulling, reduction in vision, warmth on the skin.

**Skin irritation**

An excessive response to stimulation on the skin where the patch is placed, a condition of soreness or inflammation

**Skin infection or inflammation**

Dermatitis is a blanket term meaning any "inflammation of the skin" (e.g. rashes etc.). There are several different types. Usually all of them have in common an allergic reaction to specific allergens. The term may be used to refer to eczema, which is also known as Dermatitis eczema.

**Skin injury or skin mark**

Skin mark occurs due to hyperkeratosis. Hyperkeratosis is the thickening of the skin due to frequent or long pressure applied to the part of the body.

**Skin allergy to material**

Allergy is a disorder of the immune system often also referred to as atopy. Allergic reactions occur to environmental substances known as allergens; these reactions are acquired, predictable and rapid. Strictly, allergy is one of four forms of hypersensitivity and is called type I (or immediate) hypersensitivity.

**Electric shock**

The small chance of a minor electric shock when the recorder or battery is damaged or not properly used.

**Eyebrow or Face hair removal**

The adhesive elements in the Patch may cause the removal of the facial or forehead hair when pulling the Antenna.

## Related to SENSIMED Triggerfish^®^ Recorder

**Discomfort**

Wearing the recorder in the sleeve around the upper body torso may cause reduced comfort during sleeping, physical activities and/or washing.

**Electric shock**

The small chance of a minor electric shock when the recorder or battery is damaged or not properly used.

**Patient injury, burn**

Minor skin burn from the exposed battery or wires if the recorder is damaged.

**Death**

Very small chance of the battery explosion which can cause death.

## Related to the Goldmann applanation tonometer and pneumatonometer

**Eye injury**

Physical or chemical injuries of the eye can be a serious threat to vision if not treated appropriately and in a timely fashion. Eye injury can come from a blow, scratch, or foreign object).

**Allergic reaction to anesthetic drops**

Hypersensitivity to a substance can demonstrate as itching, burning, reddening or swelling.

**Itching, burning, or gritty feeling**

Belong to irritation, inflammation, infection, allergy, injury

**Blurred vision**

Lack of sharpness of vision with, as a result, the inability to see fine detail. Blurred vision can occur when a person who wears corrective lens is without them.

**Swelling**

Belongs to allergy, inflammation, infection.

**Pain**

Pain in the eye (that is not due to injury) may be described as a burning, throbbing, aching, or stabbing sensation in or around the eye. It may also feel as if there is a foreign body in the eye.

**Pink eye (conjunctivitis)**

Conjunctivitis is inflammation of the conjunctiva or thin membrane covering the white of the eye and the inner surface of the eyelid. The inflamed conjunctiva will usually make the eye appear red or pink because the tiny blood vessels that are normally within the conjunctiva are now irritated and enlarged.

**Corneal abrasion**

A corneal erosion or abrasion can occur when the cornea is scraped or injured. In these cases, there may be a loss of the corneal epithelium, the cornea's outer layer. These painful conditions quite commonly arise after a poke from a baby's fingernail or tree limbs and bushes, or vigorous rubbing of the eye. Sometimes they are caused by contact lenses.

**Eye irritation**

An excessive response to stimulation in an eye or eyelid, a condition of soreness or inflammation

**Corneal ulcers**

A corneal ulcer, or ulcerative keratitis, is an inflammatory or more seriously, infective condition of the cornea involving disruption of its epithelial layer with involvement of the corneal stroma

## Related to proparacaine

Some local irritation and stinging may occur several hours after the instillation. Instillation of proparacaine in the eye at recommended concentration and dosage usually produces little or no initial irritation, stinging, burning, conjunctival redness, lacrimation or increased winking.

Rarely, a severe, immediate-type, apparently hyperallergic corneal reaction may occur which includes acute, intense and diffuse epithelial keratitis; a gray, ground-glass appearance; sloughing of large areas of necrotic epithelium; corneal filaments and, sometimes, iritis with descemetitis.

Allergic contact dermatitis with drying and fissuring of the fingertips has been reported.

Softening and erosion of the corneal epithelium and conjunctival congestion and hemorrhage have been reported.

1. investigator endorsement page

I have read the investigational plan of this project and I confirm that it contains all information to accordingly conduct the study. I pledge to conduct the study according to the investigational plan.

I understand and will conduct the study according to the investigational plan, any approved amendments, ICH-GCP and all applicable regulatory authority requirements and national laws.

I will not deviate from the investigational plan without prior review and written approval from the Ethics committee, except where necessary to prevent any immediate danger to the subject.

I have read and understand fully the User Manual for the SENSIMED Triggerfish^®^, and I am familiar with the device and its use according to this investigational plan.

I have sufficient time to properly conduct and complete the study within the agreed trial period, and I have available an adequate number of qualified staff and adequate facilities for the foreseen duration of the study to conduct the study properly and safely.

I will ensure that any staff at my site(s) who are involved in the study conduct are adequately trained regarding the SENSIMED Triggerfish^®^, the investigational plan and their responsibilities. In the case of delegating any of my study responsibilities I will provide the sponsor with an Investigator Responsibility Delegation log.

I will enrol the first subject only after all ethical and regulatory requirements are fulfilled. I pledge to obtain written consent for trial participation from all subjects.

I know the requirements for accurate notification of adverse incidents and I pledge to document and notify such events as described in the protocol.

I pledge to retain all trial-related documents and source data as described. I will provide a Curriculum Vitae (CV) before trial start. I agree that the CV may be submitted to the responsible regulatory authorities.

Signature: Date:

__________________________________________________________________________

Print name:

________________________________________

1. study overview – schematic

| **Exam / Visit (day)** | **Baseline visit** | **Day 1** | **Day 2** |
| --- | --- | --- | --- |
| Demographics | X |  |  |
| Medical and surgical history | X |  |  |
| Pregnancy test^[[5]](#footnote-5)^ | X |  |  |
| Automated objective refraction test | X |  |  |
| Slit lamp examination | X | X | X |
| TBUT | X |  |  |
| Corneoscleral profile evaluation | X |  |  |
| Best corrected visual acuity | X | X | X |
| Topography/keratometry | X |  |  |
| Pachymetry | X | X | X |
| Tonometry (Goldmann) | X | X^[[6]](#footnote-6)^ | X |
| Tonometry (pneumatonometer) | X | X^[[7]](#footnote-7)^ | X^[[8]](#footnote-8)^ |
| Heart rate |  | X^[[9]](#footnote-9)^ | X^8^ |
| SENSIMED Triggerfish® placing |  | X |  |
| SENSIMED Triggerfish® removal |  |  | X |
| Sleep quality assessment |  |  | X |
| Visual analogue scale |  |  | X |
| Patient diary |  | Throughout IOP fluctuation recording; collection on Day 2 | |
| Concomitant medication | On an ongoing basis | | |
| Adverse events / complaints | On an ongoing basis | | |

1. After rising from wake to sleep state IOP flattens out, so that a quadratic term is appropriately fit to the data. [↑](#footnote-ref-1)
2. Krakau CE. Calculation of the pulsatile ocular blood flow. Invest Ophthalmol Vis Sci 1992; 33: 2754-6. [↑](#footnote-ref-2)
3. Kotecha, A. et al. Intraocular pressure measurement precision with the goldmann applanation dynamic contour, and ocular response analyzer tonometers. Ophthomology 2010, in Press. [↑](#footnote-ref-3)
4. After rising from wake to sleep state IOP flattens out, so that a quadratic term is appropriately fit to the data. [↑](#footnote-ref-4)
5. For women of childbearing potential [↑](#footnote-ref-5)
6. 1^st^ eye randomised prior to SENSIMED Triggerfish installation. [↑](#footnote-ref-6)
7. IOP OU; During SENSIMED Triggerfish recording, every 2 hours in the sitting position in the wake period, 30 minutes after bedtime and then every 2 hours in supine position. [↑](#footnote-ref-7)
8. IOP in non-contact lens eye; every 2 hours in supine position until the end of the dark-sleep period, 30 minutes after waking in the sitting position and then every two hours until 24 hours. [↑](#footnote-ref-8)
9. Three times during the sleep period, for 6 minutes. [↑](#footnote-ref-9)
